# Synthesis, spectroscopic, characterization, antimicrobial, DNA interaction, DFT and molecular docking studies of a new Cu(II)-Schiff base complex

**DOI:** 10.1038/s41598-026-44842-5

**Published:** 2026-04-28

**Authors:** Eman G. Mahmoud, Eman H. Ismail, Ayman A. Abdel Aziz, Samir A. Abdel-Latif, Sarah N. Mobarez

**Affiliations:** 1 Chemistry Department, Faculty of Science, Ain Shams University, Abbassia, Cairo, 11566 Egypt; 2 Chemistry Department, Faculty of Science, Capital University (Formerly Helwan University), Cairo, 11795 Egypt

**Keywords:** Folic acid Schiff base, Cu(II) complex, DNA binding, DFT study, NLO properties, Biochemistry, Cancer, Chemical biology, Chemistry, Drug discovery

## Abstract

A novel Schiff base (L) was synthesized via the condensation of folic acid with salicylaldehyde, and its copper (II) complex (Cu–L complex) was subsequently prepared, exhibiting notable biological activity. Comprehensive characterization of L and both bulk and nanoscale forms of Cu–L complex was conducted using elemental analysis, FT-IR, UV–Vis spectroscopy, magnetic susceptibility, mass spectrometry, gel permeation chromatography, ^1^H/^13^C NMR, thermogravimetric analysis, electron spin resonance, and transmission electron microscopy. DNA binding interactions were assessed using UV–Vis absorption, ethidium bromide displacement assay, and viscosity measurements, revealing significant binding affinity. The nano-sized Cu–L complex demonstrated enhanced antimicrobial activity and potent cytotoxic effects against MCF-7 breast cancer cells, comparable to the standard chemotherapeutic agent cisplatin. Density functional theory calculations were performed at the B3LYP/6-311G(d,p) level for L and B3LYP/6-311G(d,p)-LANL2DZ for the Cu–L complex. The theoretical results were consistent with experimental data. Nonlinear optical properties, including molecular polarizability (α) and hyperpolarizability (β), indicated improved optical responses in the synthesized compounds. Molecular docking studies revealed favorable binding of Schiff base ligand (L) and its Cu–L complex to the active sites of key bacterial protein targets, supporting their potential as therapeutic agents.

**Supplementary Information:**

The online version contains supplementary material available at 10.1038/s41598-026-44842-5.

## Introduction

By condensing primary amines with aromatic aldehydes, Hugo Schiff synthesized Schiff bases for the first time. This process produced the characteristic azomethine (–C=N–) functional group, which exhibits notable properties, such as chemical stability and reversible formation^1^.

Schiff bases are organic ligands capable of coordinating with metal ions through their imine nitrogen donor site^2^. A wide range of applications in pharmacology, electrochemistry, dye production, metal ion sensing, and antibacterial action have been shown for their metal complexes^3–5^. They also act as corrosion inhibitors, catalysts, and components in optoelectronic devices^6,7^. These compounds are of high interest because of their structural tunability and capability to produce diverse metal complexes, offering many chemical and biological applications^8^.

Folic acid is one of the many molecules utilized as precursors for Schiff bases because it includes a primary amino group, which may react with aldehydes or ketones to form a Schiff base^9^. Folic acid (Vitamin B9) is a water-soluble vitamin^10^. Structurally, folic acid consists of three parts, which are the PABA (p-aminobenzoic acid), glutamic acid, and pterin ring^11^. Folic acid is a biological heterocyclic molecule that plays a vital role in cell division, DNA synthesis, fetal development, and red blood cell health^12^, and has recently exhibited therapeutic activity against cancer cells, especially MCF-7 breast cancer cells^13,14^. Human megaloblastic anemia, neural tube malformations in fetuses, cardiac problems, and cancer can all result from folic acid deficiency^15^. Therefore, there is a general agreement that keeping one’s folate level optimal is critical for avoiding illnesses induced by low folate levels. Also, salicylaldehyde and its derivatives can be effective chelating ligands when condensed with amines in ratios of 1:1 and 2:1, resulting in bi-, tri-, and tetradentate Schiff base ligands, which are ideal for complex formation with metal ions. Many biological activities, including antifungal, antimalarial, anti-proliferative, antibacterial, anti-inflammatory, antiviral, and antipyretic properties, have been shown by these metal Schiff base complexes^16^. Cu(II) metal ion is vital in biological processes, serving as an oxygen transporter in vertebrates. It is found in enzymes and different organs, including the liver, kidney, heart, and brain, attracting interest in creating copper compounds and researching their biological function^17^. There is considerable interest in using copper complexes as chemotherapeutic agents^18^.

As an illustration, it was discovered that the copper (II) complex, which is formed from cinnamaldehyde Schiff base, has a great deal of potential as an anti-cancer agent against CNS (Central Nervous System) tumor cells^19^. Additionally, the copper complex binding to 1,10-phenanthroline and a Schiff base demonstrated significant cytotoxicity against MCF-7 breast cancer cells in comparison to normal breast epithelial cells^20^. In addition, a copper complex was successfully prepared using a Schiff base obtained from the reaction between folic acid and 3-aminoacetophenone. Investigations into the biological activity revealed that the complexes have higher biological activity against the same types of bacteria than the Schiff base alone^21^. Previous studies have shown that binuclear Cu(II) complexes often demonstrate enhanced biological activity compared to mononuclear analogues due to cooperative metal–metal effects, increased positive charge density, and improved DNA interaction capability. The enhanced cytotoxic activity observed for the nano Cu–L complex is therefore consistent with literature trends for binuclear copper systems bearing mixed O, N donor environments^22–25^.

Recent studies on structurally related Cu(II) Schiff base complexes have demonstrated a significant enhancement in biological activity upon metal coordination. The chromone-based ligands coordinate to copper(II) to form tetrahedral complexes, exhibiting notable antimicrobial activity against Gram-positive bacteria (0.007–0.25 mg/mL) and Gram-negative bacteria (0.0312–0.5 mg/mL), as well as cytotoxic effects across multiple cancer cell lines, including breast, colon, and melanoma cells^26^. Similarly, Cu(II) complexes derived from bis(N, S) bidentate ligands adopt distorted tetrahedral geometries and demonstrate strong DNA binding via intercalation, with binding constants ranging from 0.675 × 10^5^ to 8.8 × 10^5^ M^−1^, highlighting their potential for DNA-targeted applications^27^. In contrast, complexes formed from tetradentate NOON ligands adopt a square planar geometry and bind to CT-DNA through intercalation, with intrinsic binding constants of 2.144 × 10^4^–4.754 × 10^4^ M^−1^. These complexes also display significant antibacterial activity, with inhibition zones of 14–23 mm at 100 ppm and 15–27 mm at 200 ppm, demonstrating higher activity than the parent ligands and comparable to standard antibiotics^28^.

The rationale behind selecting the specific ligand and metal ion for complexation in this study was guided by both chemical and biological considerations. Folic acid was chosen as the ligand precursor due to its critical role in DNA synthesis and repair, as well as its high affinity for folate receptors, which are overexpressed in many cancer cells, thereby providing inherent biological targeting capability^29,30^. Conversion of folic acid into a Schiff base introduces azomethine (–C=N–) donor groups, enhancing metal chelation, molecular planarity, and π-electron delocalization, all of which are favorable for DNA intercalation and for stabilizing the resulting metal complex^31–35^. On the other hand, copper (II) was selected as the central metal ion owing to its redox activity, biological relevance, and well-documented ability to interact with DNA via intercalative and electrostatic modes, as well as to promote reactive oxygen species-mediated DNA cleavage^36–41^. The combination of a folate-derived Schiff base ligand with Cu(II) therefore establishes a synergistic system that integrates targeted cellular recognition, strong coordination chemistry, and DNA-binding/cleavage potential, in line with contemporary strategies in bioinorganic and medicinal chemistry for designing multifunctional metal-based therapeutics. Several techniques were employed to structurally elucidate L and its Cu–L complex, and these structures were then confirmed and supported by computational modeling using density functional theory (DFT) calculations**.** The ligand and its complex were evaluated for their biological properties, including antimicrobial activity, DNA-binding affinity, and cytotoxic effects against MCF-7 breast cancer cells. The hypothesis underlying this design is that coordination to Cu(II) enhances ligand lipophilicity and electronic delocalization, facilitating membrane permeability and DNA interaction, while the folate fragment may promote selective cellular uptake.

## Experimental

### Materials and reagents

All chemicals were of analytical reagent grade and used without further purification. Folic acid, salicylaldehyde, copper carbonate, cetyltrimethylammonium bromide (CTAB), Calf Thymus DNA (CT-DNA), *tris*(hydroxymethyl)aminomethane (Tris), and ethidium bromide (3,8-diamino-5-ethyl-6-phenylphenanthridinium bromide, EB) were obtained from Sigma Chemical Co. CT-DNA stock solutions were prepared by dissolving in Tris buffer, kept at 5 °C, and used for only four successive days. All solvents, including dimethylformamide (DMF), dimethylsulfoxide (DMSO), triethylamine, and petroleum ether, were used as received from Alfa Aesar Chemical Co.

### Instrumentation

The newly prepared compounds were analyzed using various analytical techniques, including FT-IR, ^1^H NMR, ^13^C NMR, UV–Vis absorbance spectroscopy, fluorescence spectroscopy, atomic absorption spectroscopy (AAS), electrospray ionization mass spectrometry (ESI–MS), Electron ionization mass spectrometry (EI-MS), electron spin resonance (ESR), transmission electron microscopy (TEM), and gel permeation chromatography (GPC). More details are available in the Supplementary Information.

### Synthesis of the Schiff base ligand (L)

**Scheme 1:**
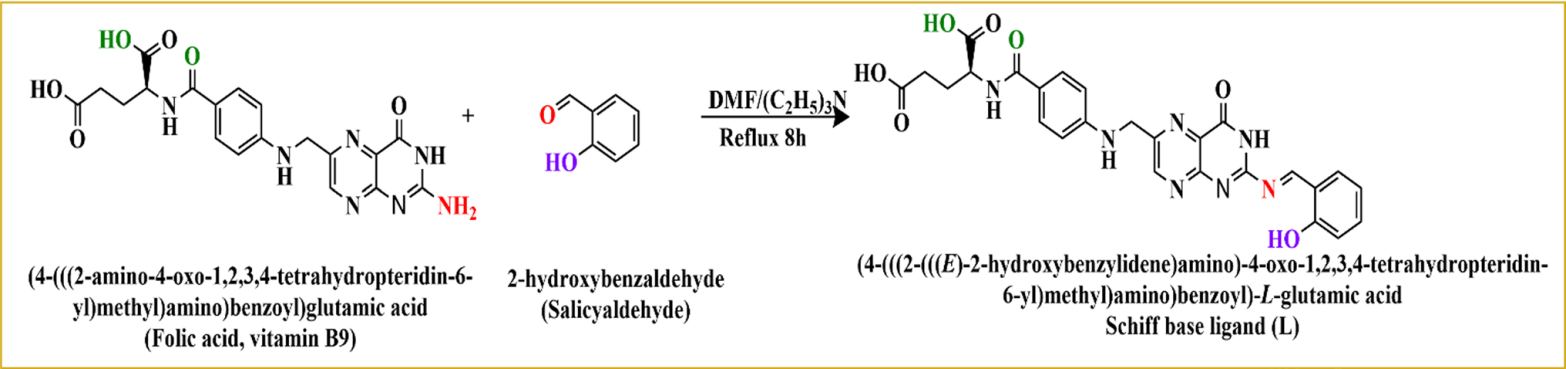
Synthetic route of Schiff base ligand (L).

Folic acid (1.986 g, 4.50 mmol) was dissolved in approximately 20 mL DMF. Salicylaldehyde (0.50 mL, 4.69 mmol, density = 1.146 g/mL) was added dropwise, followed by a few drops of triethylamine as a catalyst. The molar ratio of folic acid to salicylaldehyde was therefore approximately 1:1.04. The reaction mixture was refluxed for 8 h, leading to the formation of an orange semi-solid product. After cooling, the mixture was poured onto crushed ice to precipitate the product. The solid was filtered, washed with petroleum ether, and dried under vacuum at room temperature. The synthesis of the Schiff base ligand is schematically represented in Scheme 1.

### Synthesis of Cu–L complex

#### In the bulk scale

The ligand (0.109 g, 0.200 mmol) was dissolved in 50 mL of deionized water. The pH was adjusted to 7.0 using 0.1 M KHCO_3_ solution. CuCO_3_·Cu(OH)_2_·H_2_O (0.047 g, 0.1965 mmol) was dissolved in a minimal volume of 0.05 M HNO_3_ and added slowly to the ligand solution under stirring. The resulting metal-to-ligand molar ratio of approximately 1.97:1 was employed to ensure complete complexation. The mixture was stirred at 50 °C for 30 min. The brown precipitate formed was collected by filtration, washed with ethanol to remove residual impurities, and dried under vacuum at room temperature.

#### In the nano scale

A 3 × 10^−4^ M aqueous solution of the Schiff base ligand (L) was prepared by dissolving 0.109 g in 600 mL of deionized water. A 3 × 10^−4^ M CuCO_3_·Cu(OH)_2_.H_2_O solution was prepared separately by dissolving 0.047 g of CuCO_3_·Cu(OH)_2_·H_2_O in 600 mL of deionized water acidified with nitric acid. Equal volumes (10 mL each) of the ligand solution and CTAB solution (1 × 10^−5^ M) were mixed, followed by slow dropwise addition of 10 mL of the CuCO_3_·Cu(OH)_2_·H_2_O solution under continuous stirring at 50 °C for 30 min. The resulting brown precipitate was collected, thoroughly washed with ethanol to remove residual surfactant^42^, and dried under vacuum at room temperature. All molar concentrations, masses, and temperature values were rechecked for internal consistency. The removal of the CTAB surfactant layer was further confirmed by thermogravimetric analysis (TGA), which displayed a decomposition profile consistent with that of the bulk Schiff base–Cu(II) complex.

### Biological efficiency

#### Antimicrobial activity

Agar well diffusion method^43^ was employed to evaluate the antimicrobial activity of the Schiff base ligand (L) and its Cu–L nano complex. The detailed approach is provided in the Supplementary Information.

#### DNA binding experiments

The detailed study of the in vitro CT-DNA interaction with the Schiff base ligand (L) and its Cu–L complex is explained in the Supplementary Information.

#### Cytotoxicity studies

The evaluation of the cytotoxic effect of the Schiff base ligand (L) and the nanosized Cu–L complex on breast cancer cells was done using the methylthiazol tetrazolium (MTT) assay. MCF-7 cells (ATCC No. HTB-22™ human breast cancer cell line) were obtained from American Type Culture Collection (ATCC; Rockville, MD, USA). The cell lines were propagated according to ATCC guidelines. All anticancer assays were conducted in the tissue culture unit at the Regional Center for Mycology and Biotechnology, Al-Azhar University, Cairo, Egypt. The detailed studies were clarified in the Supplementary Information.

### Computational details

Since the single-crystal X-ray method for determining the precise geometric structure^44^ wasn’t accessible, we relied on computational analysis for energy minimization and structural illustration. The geometrical conformations of the synthesized compounds in their gas-phase ground state were optimized using the density functional theory (DFT) approach at the B3LYP functional level, implemented through the Gaussian-09W software package^45–48^. The solid Cu–L complex structure was optimized using the metal ion basis set 6-311G(d,p)-LANL2DZ^49–53^, whereas for the free ligand, the B3LYP/6–311G(d,p) level was used^51,54,55^. Geometry optimization was performed to fully optimize all bond lengths, dihedral angles, and bond angles. The DFT theory may be used to study many factors, such as the geometrical variables, the energy optimization, and the three-dimensional MEP maps. The quantum chemical variables were demonstrated, and the Schiff base ligand (L) and its Cu–L complex chemical reactivity were assessed based on computations^56,57^. MO schemes have been established according to a Gauss View 5^58^ program. Calculations were made utilizing the x, y, and z coordinates to determine the nonlinear optical characteristics of the prepared compounds, specifically Δα, μ, <β>, and <α>.

### Molecular docking

Molecular docking was investigated using the MOE2022 program^59^. The protein crystal structures of the bacteria *Aspergillus niger* (PDB ID: 4C7A), *Candida albicans* (PDB ID: 3QLW), *Escherichia coli* (PDB ID: 5I39), and *Staphylococcus aureus* (PDB ID: 3Q8U) were identified using the Protein Data Bank (PDB). Conformational shapes were chosen using the calculated binding scores, E-conformation values, and the perfect binding of the studied chemicals to the associated amino acids of protein receptors, throughout numerous docking simulations performed under default parameters.

## Results and discussion

This work presents the preparation, characterization, investigation of biological effectiveness, and computational studies of a novel copper (II) chelate derived from a novel Schiff base ligand (L), which was prepared via condensation between folic acid and salicylaldehyde. The structures of the Schiff base ligand (L) and its Cu(II) complex were established using FT‐IR, ^1^H NMR, ^13^C NMR, ESI–MS, and microanalysis. The elemental analysis data of the Schiff base ligand (L) and its bulk Cu–L complex are presented in Table 1. The experimentally determined percentages of C, H, N, and Cu were in good agreement with the calculated values, confirming the proposed stoichiometry. The ligand was found to be soluble in water, whereas the Cu(II) complex was insoluble in common organic solvents and freely soluble in DMSO.

**Table 1:** Elemental analysis of the prepared Schiff base ligand (L) and its bulk Cu–L complex.

Compound	M.Wt	C %	H %	N %	Cu %
		Cal. (Found)			
L C_26_H_23_N_7_O_7_	545.50	57.25 (57.23)	4.25 (4.60)	17.97 (17.1)	–
Bulk Cu–L [C_26_H_29_Cu_2_N_7_O_11_]·8H_2_O [C_26_H_45_Cu_2_N_7_O_19_]	886.76	35.22 (33.98)	5.11 (4.56)	11.06 (13.42)	14.33 (15.00)

### FT-IR spectroscopy

**Fig. 1:**
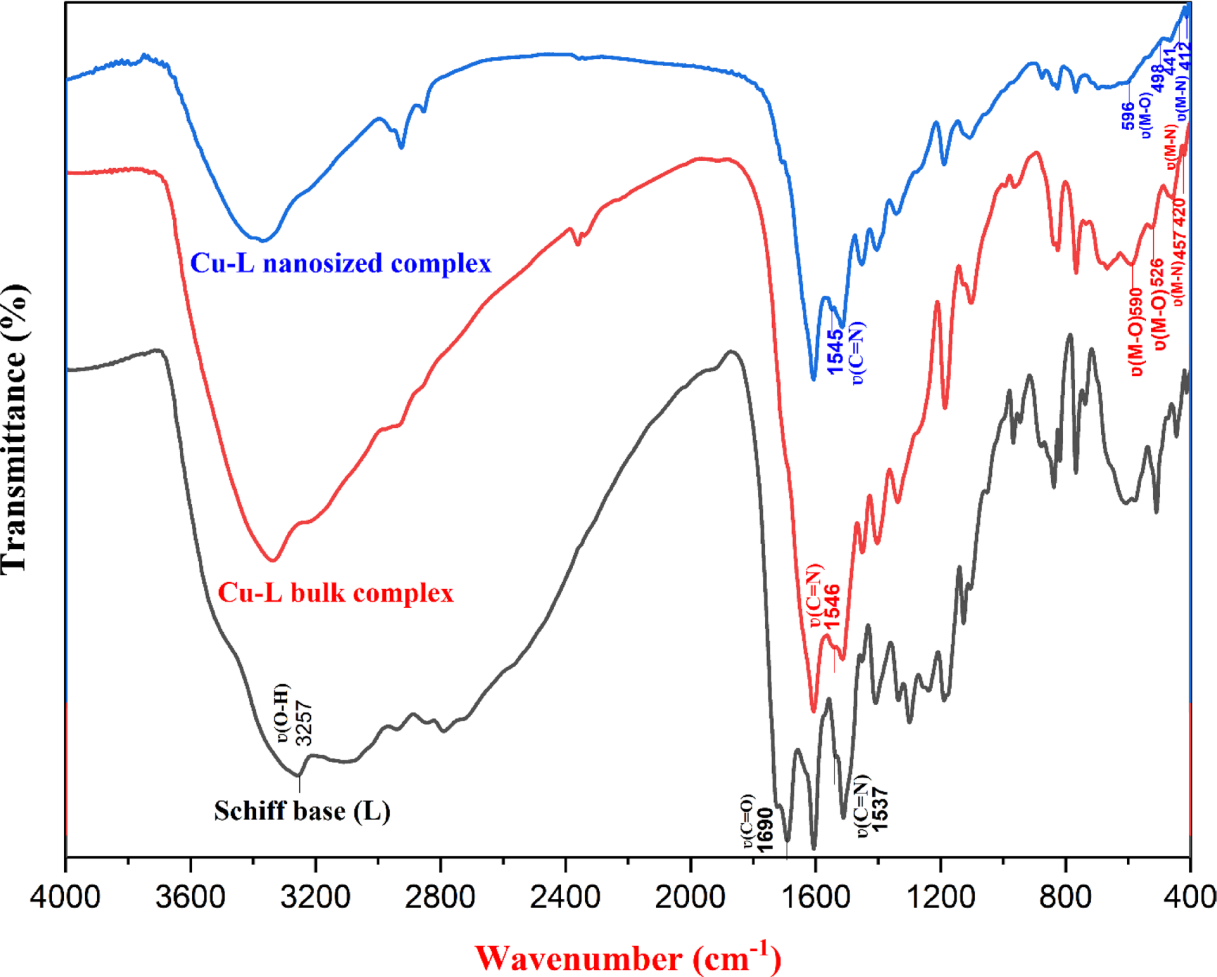
FT-IR spectra of the Schiff base ligand (L) and its Cu–L complex in bulk and nanosized dimensions.

Table S1 summarizes the most relevant IR vibrational bands of the Schiff base ligand (L) and its Cu–L complex with their respective assignments. The IR spectra show considerable peak overlap due to the presence of multiple functional groups in the Schiff base ligand (L) and its Cu–L complex in both bulk and nanosized forms. Therefore, almost all of the bands overlap with each other. The FT-IR spectra for Schiff base ligand (L) and its Cu–L complex in bulk and nano-sized forms are shown in Fig. 1.

The FT-IR spectrum of the free ligand (L) exhibited a characteristic band at 1537 cm^−1^^60,61^ assigned to the azomethine ν(C=N) stretching vibration. The primary amine bands of folic acid at 3322 and 3415 cm^−1^, ascribed to the symmetric and asymmetric N–H stretching vibrations, disappeared after condensation, confirming Schiff base formation. Additionally, a new band at 3257 cm^−1^ appeared, corresponding to the O–H stretching vibration of the phenolic group in the salicylaldehyde moiety^62^. Furthermore, the Schiff base ligand (L) showed a prominent absorption band at 1690 cm^−1^, attributed to the free carbonyl (C=O) stretching vibration of the carboxylic group^63^.

In the Cu–L complex, the disappearance of the carbonyl band suggests involvement of the carboxylate oxygen in coordination. In addition, the ν_(C=N)_ band shifted to lower wavenumbers, which indicates coordination through the imine nitrogen atom. The Far-IR spectra of the Cu–L complex showed the most prominent evidence of complexation, with low-frequency bands attributed to Cu–O and Cu–N stretching vibrations appearing at 590–420 cm^−1^ for the bulk complex and at 596–412 cm^−1^ for the nanosized complex, respectively. Additionally, broad bands at 3224 cm^−1^ (bulk) and 3268 cm^−1^ (nanosized) were observed, ascribed to the coordinated water molecules’ O–H stretching vibrations.

### UV -VIS absorption spectroscopy

**Fig. 2:**
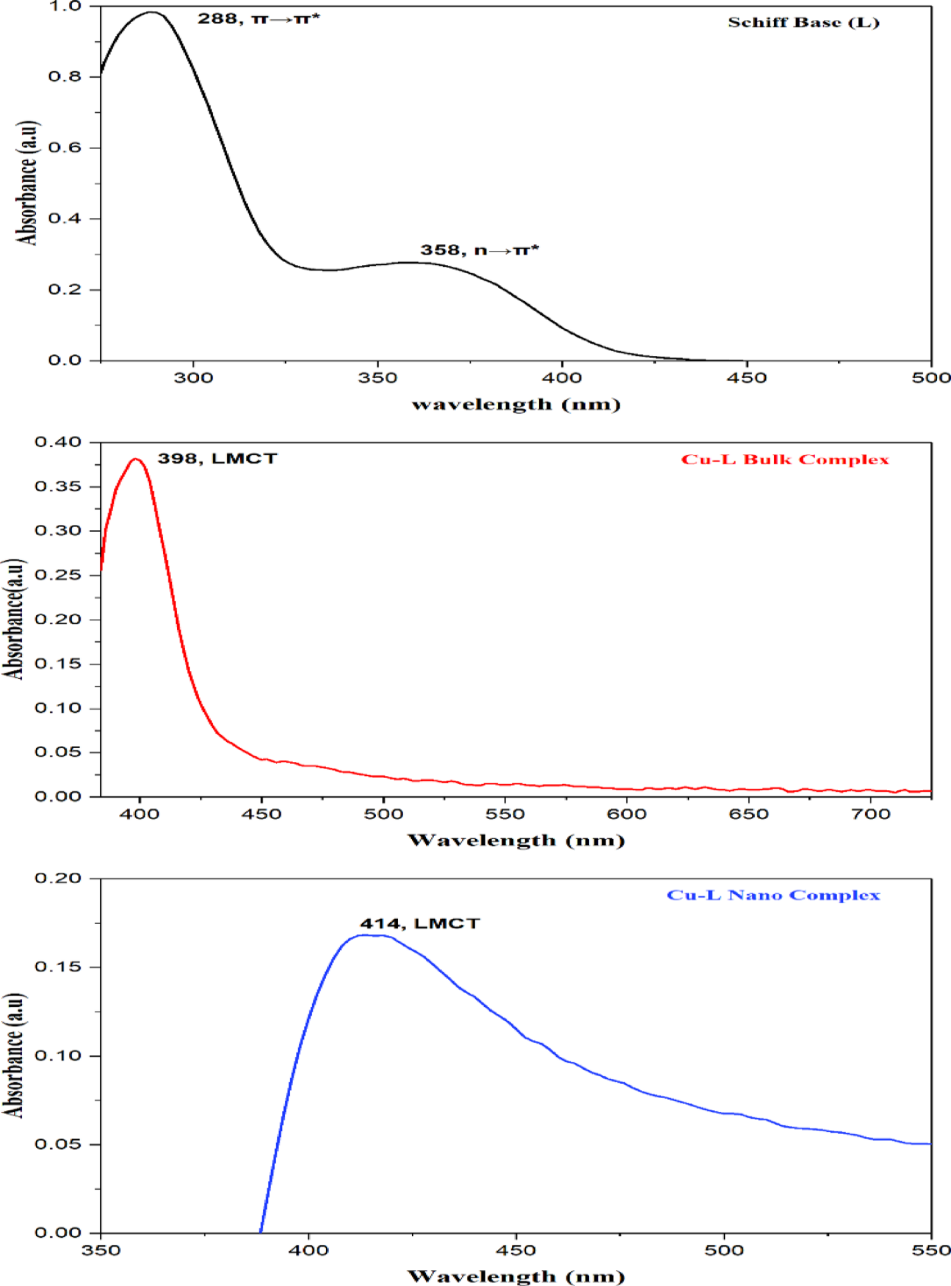
The UV- Vis absorption spectra of Schiff base ligand (L), Cu–L bulk complex, and Cu–L nanosized complex.

In this study, electronic spectra were utilized to suggest the stereochemistry of the Cu ion in bulk and nanosized complexes by analyzing the number and positions of the transition bands. The spectra of the free ligand and its Cu–L complex in both bulk and nanosized dimensions were obtained at room temperature in the 300–800 nm range using DMSO as the solvent (Fig. 2). The electronic spectrum of the ligand displayed a prominent absorption band at 358 nm attributed to an n → π* transition. Upon complexation, new bands appeared at 398 nm (bulk) and 414 nm (nano), assigned to ligand-to-metal charge transfer (LMCT) transitions^64^.

### Magnetism and ESR study

The Cu–L complex exhibits a magnetic moment of 3.3126 B.M., indicating the presence of multiple unpaired electrons and supporting a binuclear copper structure. This value exceeds the expected spin-only moment for a single Cu(II) ion, suggesting magnetic coupling between the two copper centers^65^. Electron spin resonance (ESR) spectroscopy is a valuable technique for studying the local environments and structures of paramagnetic species. Cu(II) ESR anisotropic spectrum of the Cu–L complex is captured in the polycrystalline state at room temperature (Fig. S1). The complex’s ESR spectrum exhibits a single anisotropic broad signal with ${\mathrm{g}}_{\perp }$ > ${\mathrm{g}}_{\parallel }$ (${\mathrm{g}}_{\parallel }$= 2.09057 and ${\mathrm{g}}_{\perp }$= 2.20442), suggesting a ${d}_{{Z}^{2}}$ ground state, which may indicate a less common compressed octahedral or possibly a distorted tetrahedral geometry surrounding the Cu(II) center^66^. From these, the g_avg_-value (${\mathrm{g}}_{\mathrm{avg}}= \frac{{\mathrm{g}}_{\parallel }+ 2{\mathrm{g}}_{\perp }}{3}$) was calculated to be approximately 2.1665, and the g-anisotropy (${\Delta }_{\mathrm{g}}$ = ${\mathrm{g}}_{\perp }-{\mathrm{g}}_{\parallel }$) was found to be 0.11385, indicating significant deviation from isotropy.

The axial symmetry parameter (R), calculated as $=\frac{{\mathrm{g}}_{\parallel }-2.0023}{{\mathrm{g}}_{\perp }-2.0023}$, yielded a value of 0.4367. Since R < 1, this suggests a ${d}_{{z}^{2}}$ ground state for the unpaired electron, which is in agreement with the observed trend ${\mathrm{g}}_{\perp }$> ${\mathrm{g}}_{\parallel }$^67^. These ESR parameters collectively support the assignment of a distorted four-coordinate geometry, likely a tetrahedral structure, around the Cu(II) center^68^. Furthermore, the G-value, a measure of exchange interaction between Cu(II) centers, was also calculated as 0.4367 using the same expression, which is notably less than the critical value of 4. This low G-value indicates strong magnetic exchange coupling between copper centers, supporting the existence of a binuclear Cu(II) complex with significant intermetallic interaction and a distorted tetrahedral geometry^69^.

### Mass spectrometry

**Fig. 3:**
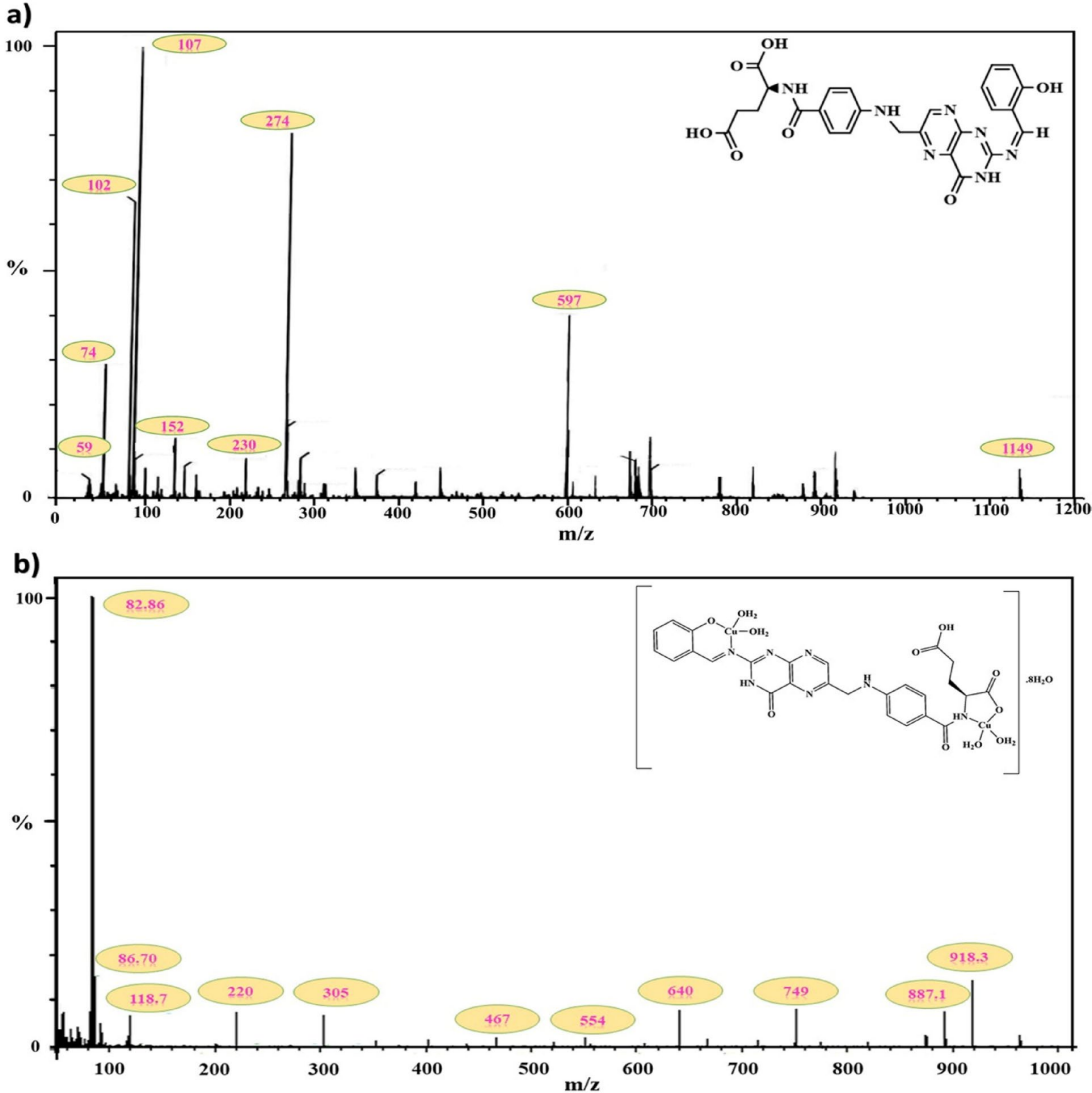
Mass spectrum for (**a**) Schiff base ligand (L) and (**b**) Cu–L complex.

Mass spectrometry is a dependable technique for determining the molecular and chemical structures of the produced Schiff base ligand (L) and its Cu–L complex. The mass spectrum of the Schiff base ligand (L) (Fig. 3) exhibited a molecular ion peak at m/z = 1149, significantly higher than the calculated monomer molecular weight of 545.50 g/mol, suggesting the presence of a dimeric form^70,71^. A distinct peak at m/z = 597 further confirmed the formation of the monomeric species with the molecular formula C₂₆H₂₃N₇O₇. Further fragment ion peaks and their proposed assignments are summarized in the subsequent Table S2. The mass spectrum of the Cu–L complex (Fig. 3) exhibits a molecular ion peak extending beyond m/z = 918.29 g/mol, which is higher than the calculated molecular weight of the monomer (886.76 g/mol). This indicates the possible presence of a polymeric form of the complex, in agreement with the GPC results. In addition, a distinct peak at m/z = 887.1 g/mol confirms the existence of the monomeric Cu–L structure.

### ^1^H/^13^C NMR spectroscopy

**Fig. 4:**
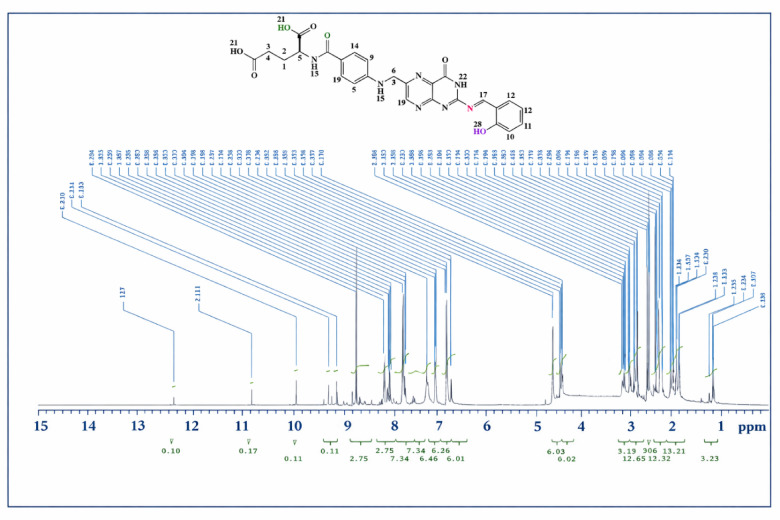
^1^H-NMR spectrum of Schiff base ligand (L).

**Fig. 5:**
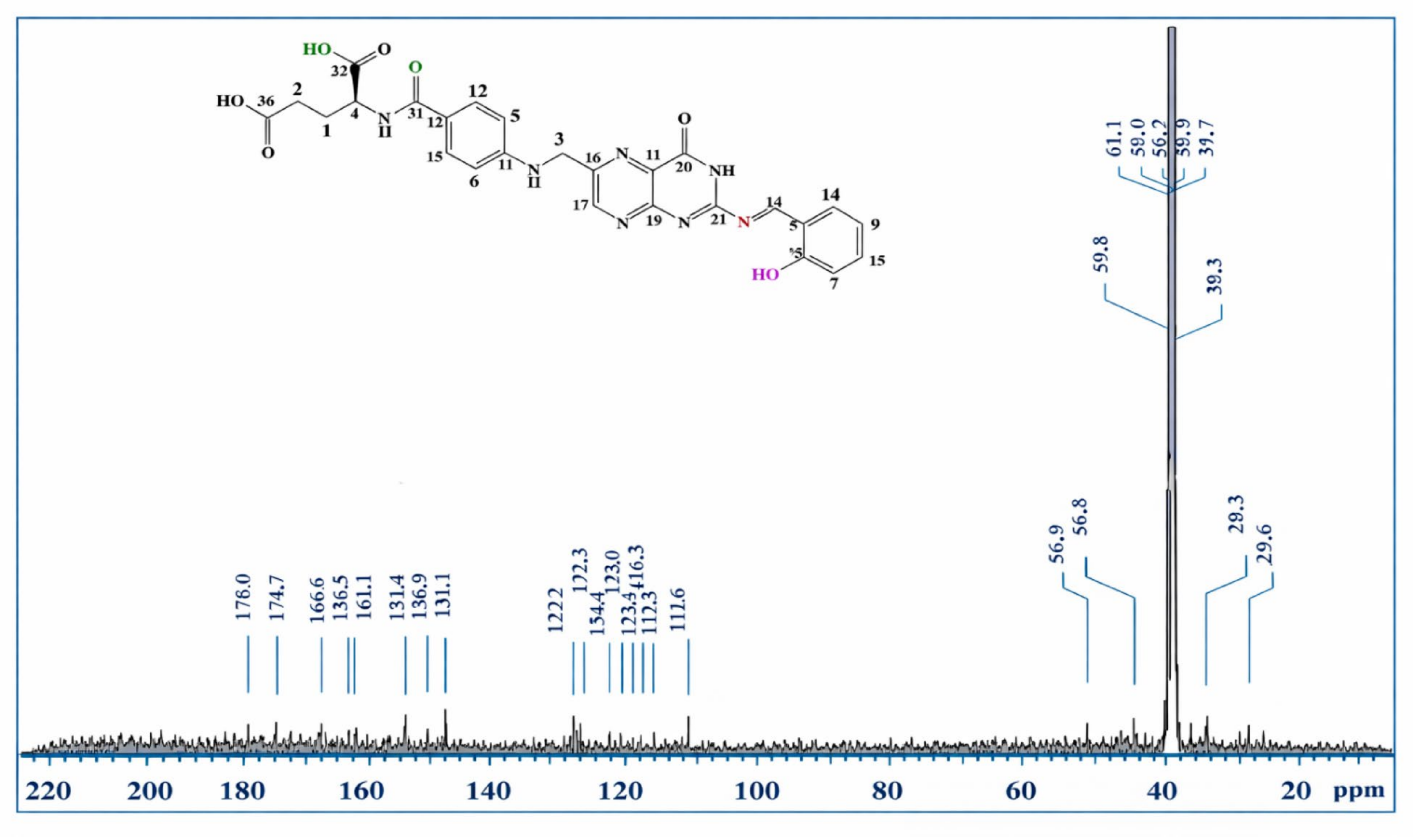
^13^C NMR spectrum of Schiff base ligand (L).

The recorded ^1^H NMR and ^13^C NMR spectra of the Schiff base ligand (L), obtained in deuterated dimethyl sulfoxide (DMSO-d₆), exhibited distinct proton and carbon resonances consistent with the proposed molecular structure. The synthesis of the Schiff base was confirmed by a singlet signal at δ 8.62 ppm in the ^1^H NMR spectrum (Fig. 4), corresponding to the azomethine (–CH=N) proton. Two additional singlets at δ 10.84 and 12.7 ppm were attributed to phenolic protons (H_2_O and H21,23). The presence of an amide group (–NH) was supported by three singlet peaks at δ 7.08, 8.67, and 12.7 ppm. Moreover, the spectrum displayed triplet signals at δ 2.28, 4.28, and 6.90 ppm attributed to protons H3,4, H5, H11,12, and a quartet at δ 1.89 ppm corresponding to H1,2. Additional singlet peaks were observed at δ 4.41, 8.62, and 9.03 ppm, assigned to protons H6,7, H18, and H19, respectively. A comprehensive summary of the chemical shift assignments is provided in Table S3. The ^13^C NMR spectrum of the Schiff base ligand (L) (Fig. 5) exhibited a resonance at δ 162.5 ppm, corresponding to the azomethine carbon (C=N). A detailed overview of the assigned carbon chemical shifts is presented in Table S4.

### Gel permeation chromatography

Gel permeation chromatography (GPC), a variant of size exclusion chromatography (SEC), was employed to determine the molecular weight distribution of the Cu–L complex. This technique separates analytes based on their hydrodynamic volume, making it particularly effective for analyzing polymers. The GPC chromatogram of Cu–L bulk complex (Fig. S2) showed a broad peak at a retention volume of 12 mL, which corresponds to the most abundant species in the sample. The number average molecular weight (M_n_) was 58,156 g/mol, and the weight average molecular weight (M_w_) was 138,357 g/mol. From these values, the polydispersity index (PDI) was calculated using the relation:

1 $$\frac{\mathrm{Mw}}{\mathrm{Mn}}=PDI= \frac{138357}{58156}\approx 2.38$$

However, the GPC software reported (PDI = 2.27), which is slightly lower than the manual calculation. This difference can be explained as documented in the literature by the way the instrument software processes the chromatogram. The software often applies baseline corrections and excludes small parts of the peak tails or noise, which reduces the apparent polydispersity. In contrast, the manual calculation uses only the ratio between Mw and Mn without any corrections^72^. Despite the small variation, both values (calculated and reported PDI) indicate a broad molecular weight distribution, reflecting the presence of both shorter and longer polymeric chains^73^. These results suggest the likelihood of intermolecular hydrogen bonding between Cu–L monomeric units, leading to the formation of a polymeric structure.

### Thermogravimetric analysis (TGA) studies

Figure S3 presents the thermogravimetric (TG) and derivative thermogravimetric (DTG) profiles of the synthesized Schiff base ligand (L) and its Cu–L complex in both bulk and nanoscale forms, obtained under a nitrogen atmosphere at a heating rate of 10 °C/min. This study revealed a high consistency between theoretical and experimental mass reductions, supporting the proposed structure of the Schiff base ligand (L) and Cu–L complex as shown in Table S5. The ligand decomposed in three stages between 40 and 1000 °C with total mass loss consistent with calculated values. The first stage, occurring between 40 and 219 °C, shows a mass loss of 11.35% (Calc. 11.19%), indicating the liberation of carbon dioxide and ammonia gas. The second decomposition step, taking place from 219 to 303 °C, involves a weight reduction of 18.56% (Calc. 19.27%), indicating the loss of NH_3_, CO_2_, C_2_H_4_, and CH_4_. The final degradation phase occurs between 303 and 1000 °C, with a mass loss of 62.74% (Calc. 62.93%), due to the removal of the organic moiety C₁₈H₉N₅O₃. Overall, the Schiff base ligand exhibits a total weight loss of 92.65% (Calc. 93.39%), leaving three carbon atoms as residue.

The thermogram for the Cu–L complex in bulk size demonstrated four decomposition phases. The first stage occurred between 48 and 154 °C, showing a mass loss of 12.02% (Calc. 12.19%), which corresponds to the release of six hydration water molecules. The second decomposition phase was accompanied by a 5.76% (Calc. 5.98%) weight loss in the temperature range of 154–208 °C and may have involved the loss of the two crystalline water molecules and ammonia gas. The third stage was characterized by the loss of 4H_2_O (coordinated), CO_2_, and C_2_H_4_ in the temperature range of 208–357 °C. At a temperature range of 357–887 °C, the final stage took place, resulting in a weight loss of 47.25% (Calc. 47.63%), which is equivalent to the organic portion, C_23_H_14_N_6_O_3_, leaving behind a residue of two CuO units.

In the nano-sized Cu–L complex, degradation occurs in three distinct stages. In the first stage, occurring between 38 and 149 °C, a mass loss of 10.2% (Calc. 10.16%) was recorded, corresponding to the removal of five crystalline water molecules. The second stage, observed within the 149–268 °C range, showed a weight reduction of 15.5% (Calc. 16.14%), which can be attributed to the elimination of three lattice water molecules, four coordinated water molecules, and ammonia. During the third stage, which happened between 268 and 519 °C, the organic part of C_22_H_18_N_6_O_5_ was lost, leading to a weight loss of 50.76% (Calc. 50.34%). The calculated value of 76.64% was nearly met by the measured total weight loss of 76.54%, leaving two CuO units contaminated with four carbon atoms as a residue.

### Transmission electron microscopy (TEM)

**Fig. 6:**
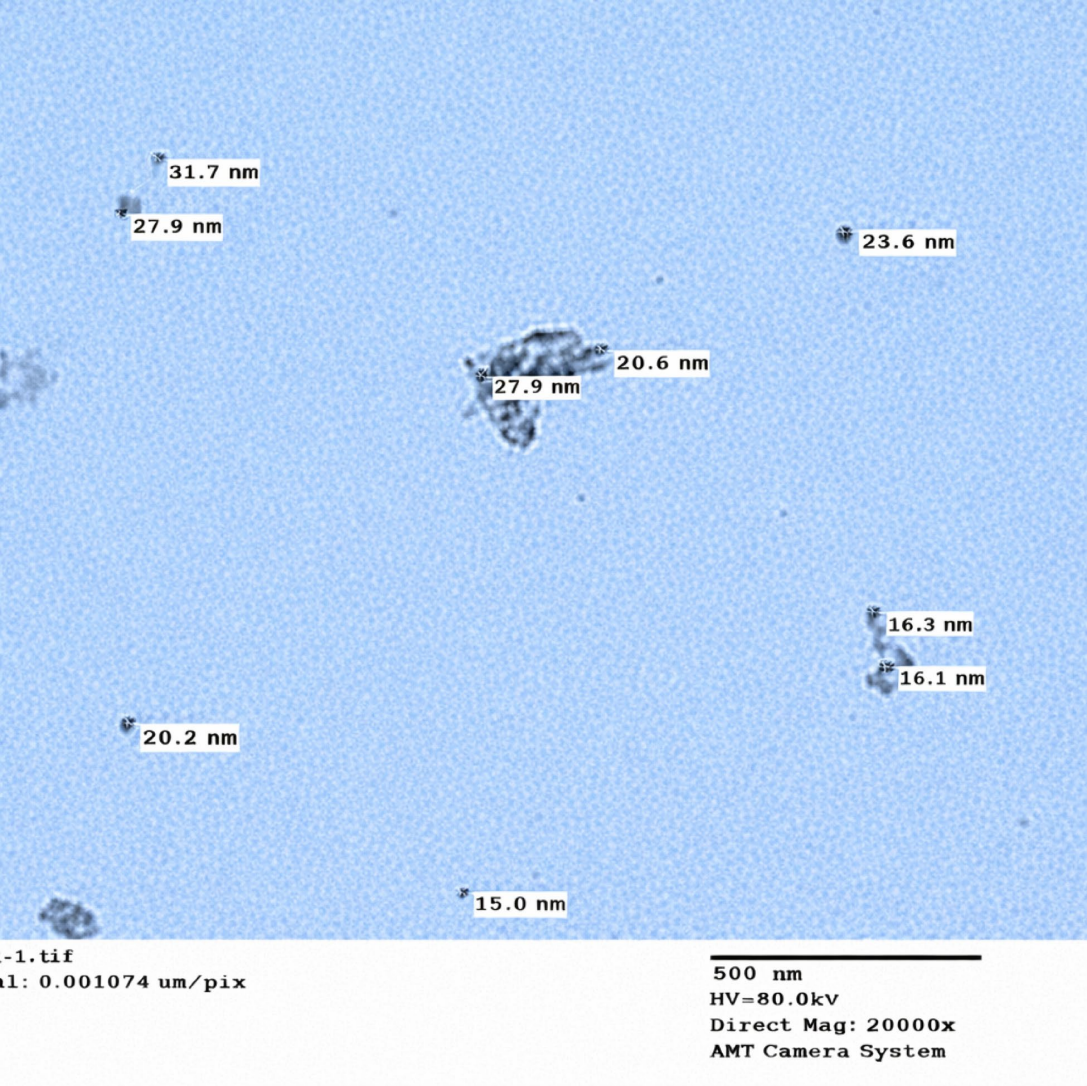
TEM image of Cu–L nano complex.

Transmission electron microscopy (TEM) was employed to investigate the morphological characteristics and particle size distribution of the synthesized Cu–L nano complex. As illustrated in Fig. 6, TEM analysis of the Cu–L nano complex revealed well-defined nanoparticles with sizes ranging from 15 to 27 nm. These results confirm the successful formation of the complex within the nanometric scale.

**Scheme 2:**
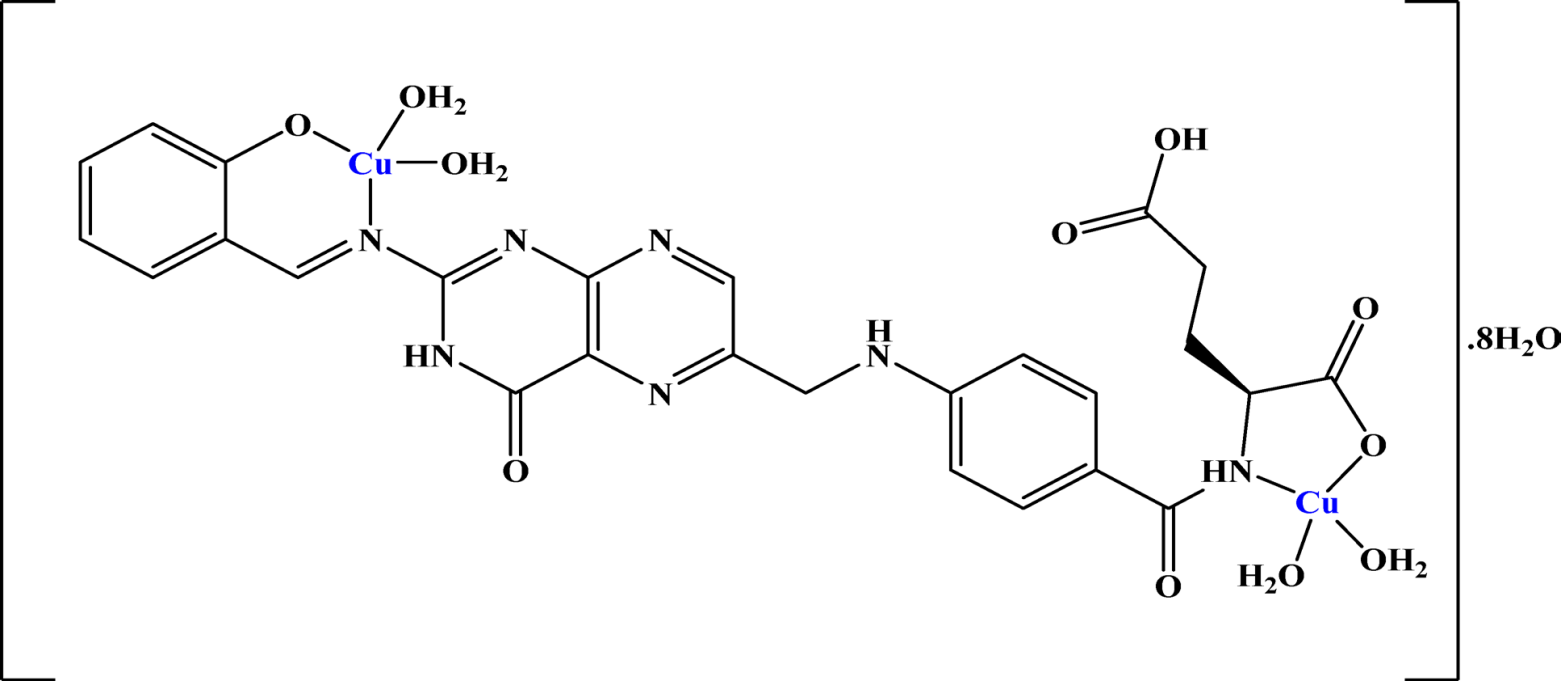
The proposed structure of Cu–L complex.

Based on analytical, magnetic moment, spectral, and thermal data, a tetrahedral geometry has been suggested for Cu(II), which is depicted in Scheme 2.

### Biological evolution

#### Antimicrobial activity

**Fig. 7:**
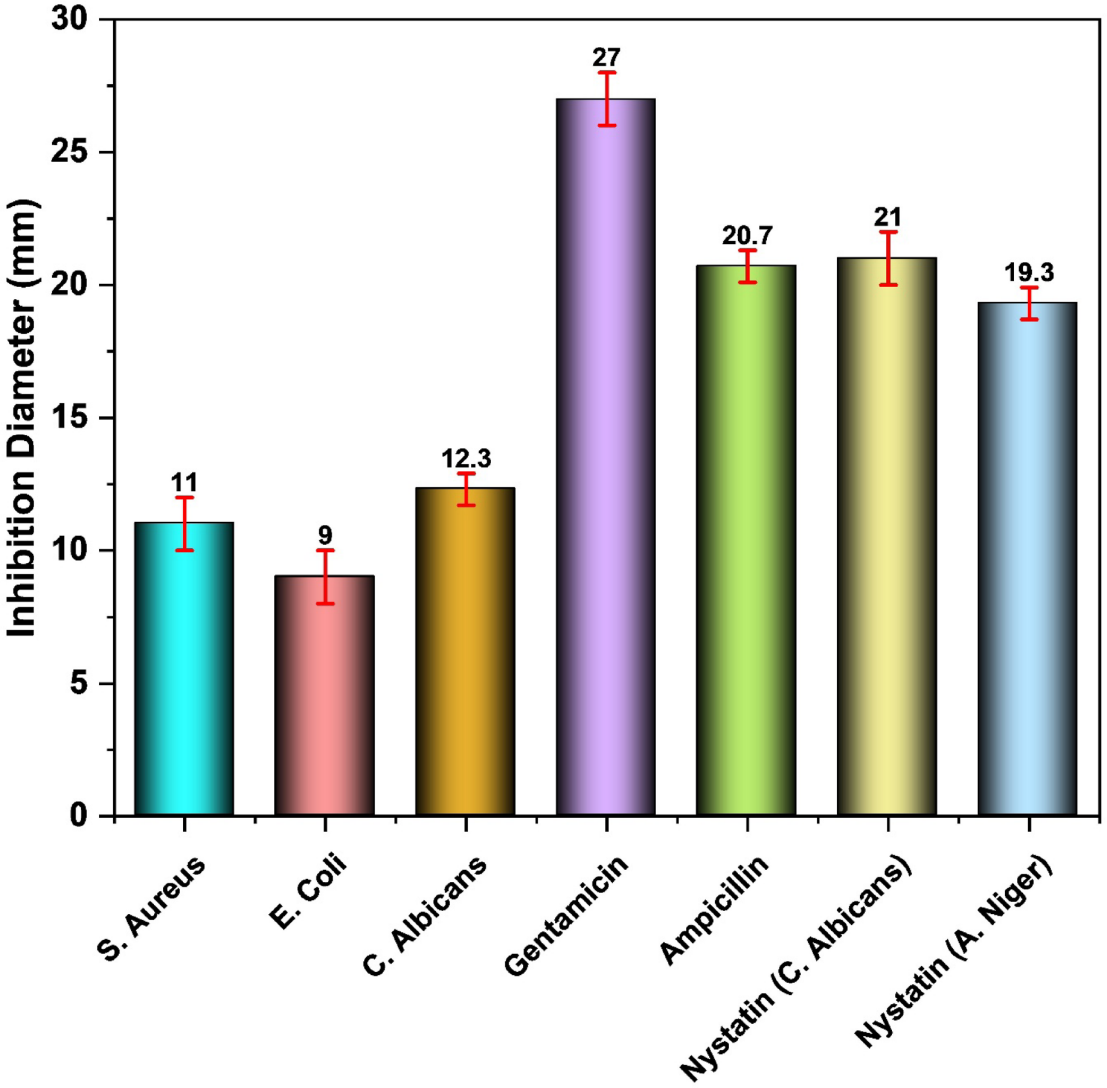
Antibacterial and Antifungal Activities of Schiff Base ligand (L) and its Metal Cu(II) Complex.

The in vitro antimicrobial potential of the Schiff base ligand and its Cu–L nano complex was assessed against bacterial isolates *Staphylococcus aureus* (ATCC 13565), and *Escherichia coli* (ATCC 10536) using Ampicillin and Gentamicin as controls. The antifungal activity of the Schiff base and Cu–L nano complex was carried out against fungal isolates *Candida albicans* (ATCC 10231) and *Aspergillus niger* (ATCC 16404) using Nystatin as a control. The selection of *A. niger* (PDB ID 4C7A)*, C. albicans* (PDB ID 3QLW)*, E. coli* PDB ID 5I39)*, and S. aureus* (PDB ID: 3Q8U) as protein targets is based on their representative clinical importance and widespread use as model organisms in antimicrobial research^74–77^. *A. niger* and *C. albicans* are opportunistic fungal pathogens responsible for significant morbidity in immunocompromised patients, with *C. albicans* being one of the most prevalent causes of invasive mycoses in humans and a frequent subject of in silico and in vitro target validation studies due to the limited efficacy of existing antifungals^74,75^. Similarly, *E. coli* and *S. aureus* are ubiquitous bacterial pathogens that serve as prototypical Gram-negative and Gram-positive organisms in antimicrobial drug discovery, which particularly due to their prevalence in urinary, gastrointestinal, and systemic infections—and their well-characterized protein structures in the Protein Data Bank that facilitate reliable docking analysis^76,77^. Moreover, multiple studies have demonstrated that molecular docking against proteins from these microorganisms can correlate with antimicrobial activity and mechanism elucidation; for example, targeting essential enzymes and virulence factors in *E. coli* and *S. aureus* has been shown to predict antibacterial potential of compounds in silico prior to experimental validation^76,77^. In the case of fungal targets, docking against *A. niger* and *C. albicans* proteins has been widely used to investigate antifungal binding interactions of endogenous and exogenous metabolites^74,75^. Therefore, these protein structures were chosen not only for their availability of high-resolution crystallographic data but also for their biological relevance as targets in antimicrobial and antifungal drug research, allowing evaluation of potential broad-spectrum activity across both bacterial and fungal pathogens. The antibacterial results (Fig. 7) showed that the Cu–L nano complex exhibited greater inhibitory activity than the free Schiff base ligand, which showed no antimicrobial effect against the four tested bacterial strains. The enhanced activity of the Cu–L complex is attributed to increased lipophilicity and improved membrane permeability upon Cu(II) coordination. Regarding antifungal activity, the Cu–L nano complex was active against *C. albicans,* while no inhibition was observed against *A. niger*.

#### DNA binding

The formation of a 2:1 metal‑to‑ligand stoichiometry allows the Cu(II) ions to adopt a tetrahedral coordination, which minimizes steric hindrance and maximizes orbital overlap with the azomethine nitrogen and phenolic oxygen donors of the Schiff base^78^. This geometry also promotes planarity and extended π‑conjugation, which enhances π–π stacking interactions with DNA and improves antimicrobial and cytotoxic efficacy^37,79^. This binuclear tetrahedral arrangement also influences the electronic distribution around copper centers, enhancing ligand‑to‑metal charge transfer (LMCT) and promoting moderate DNA intercalation^78,80^. The cooperative presence of two Cu(II) centers likely contributes to improved biological activity through increased electrostatic interaction with negatively charged DNA phosphate backbones^37,79^. Thus, the observed biological properties can be directly correlated with the coordination environment, nuclearity, and electronic characteristics of the complex^37,78,80^. These structural features are consistent with previous reports showing that tetrahedral or distorted‑tetrahedral geometries in Cu(II) Schiff base complexes favor both stability and bioactivity^37,78,80^.

##### Absorption spectroscopic studies

**Fig. 8:**
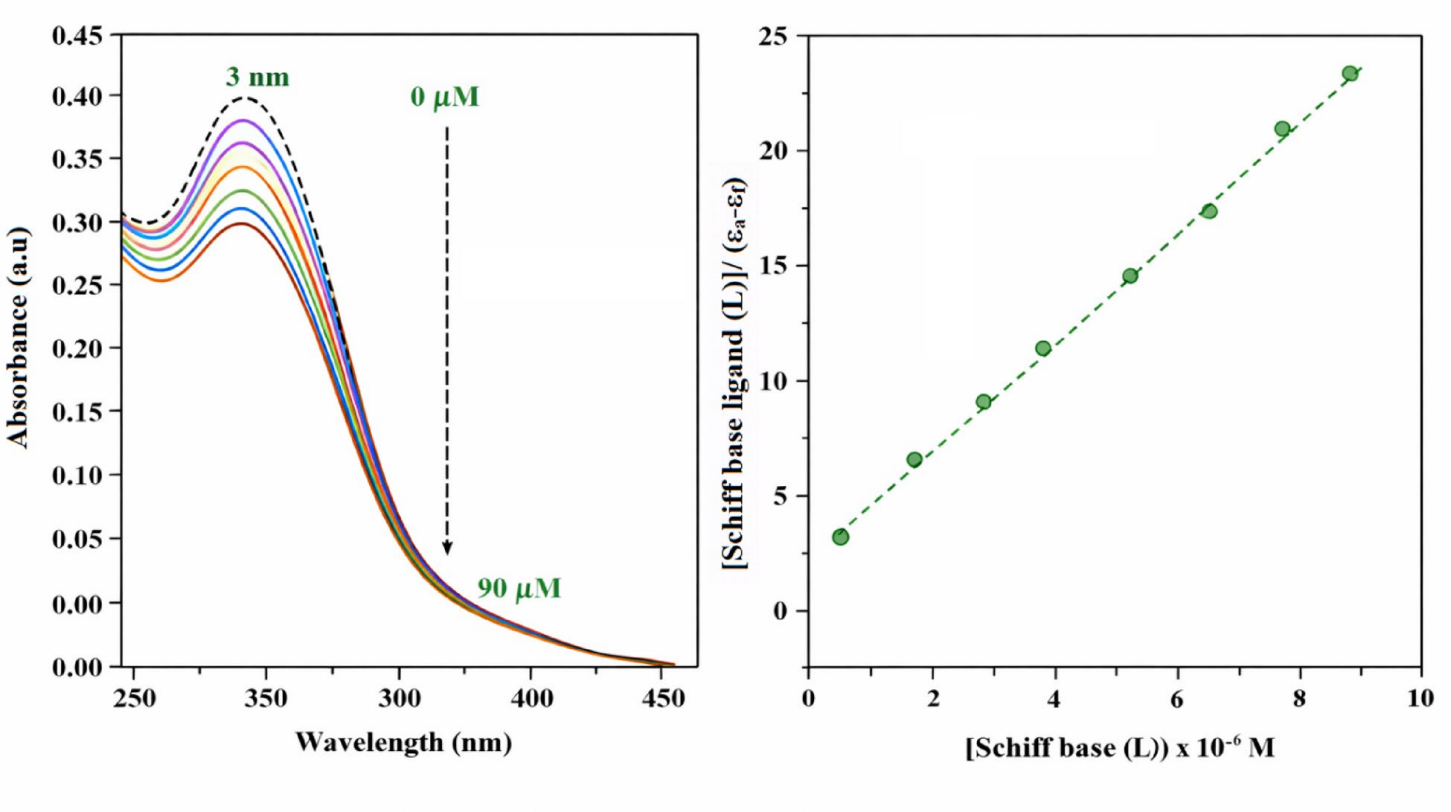
Absorption spectra of CT-DNA (10 μM) in the absence (dashed line) and presence (solid line) of Schiff base ligand (L) (0–90 μM) at room temperature in 5 mM Tris–HCl–NaCl buffer (pH 7.4) and plot of [Schiff base ligand (L)/(ɛ_a_ − ɛ_f_) vs. [Schiff base ligand (L)].

**Fig. 9:**
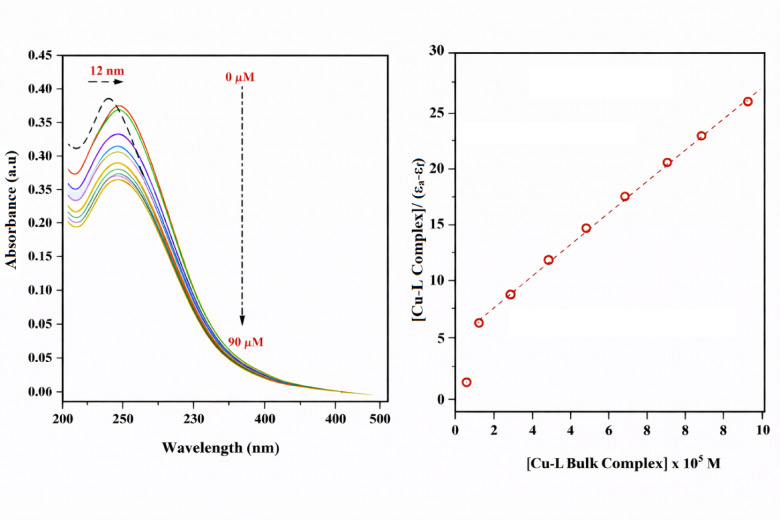
Absorption spectra of CT-DNA (10 μM) in the absence (dashed line) and presence (solid line) of Cu–L bulk complex (0–90 μM) at room temperature in 5 mM Tris–HCl–NaCl buffer (pH 7.4) and plot of [Cu–L bulk complex]/(ɛ_a_ − ɛ_f_) vs. [Cu–L bulk complex].

UV–absorption spectroscopy, which is a very common technique to study the binding mode of DNA with small molecules^81^ was used to investigate the binding affinity of the Schiff base ligand (L) and its complex to calf thymus DNA (CT-DNA). The absorption spectra of a fixed concentration of CT-DNA, with and without the Schiff base ligand (L) and Cu–L complex, are displayed in Figs. 8 and 9, respectively. The degree of hypochromism indicates the strength of the intercalative bond^82^. The hypochromic effect is generally caused by the electronic states of the compound interacting with those of the DNA bases^83^, whereas the red shift is attributed to the reduction in the energy gap between the HOMO and LUMO orbitals, due to binding of the compounds with DNA^84^. Hyperchromism has been noticed when various medications interact with DNA^85^. This effect may indicate either an external contact (electrostatic binding^86^) or partial uncoiling of the DNA helix, which exposes additional bases of the DNA^87^. The addition of ligand (L) and Cu–L complex to CT-DNA caused hypochromism and bathochromic shifts. The ligand (L) caused a 3 nm red shift of the absorption band at 280 nm, while the Cu–L complex caused a more pronounced 12 nm red shift. Both compounds also induced a notable hypochromism for this band. This observation proves the intercalation of the reported complex by stacking and interacting with the ligand’s aromatic rings with the DNA base pairs^88^. A plot of [Schiff base ligand (L)] or [Cu–L Complex]/(ε_a_*–*ε_f_*)* versus [Schiff base ligand (L)] or [Cu–L complex] gave a straight line, as shown in Figs. 8 and 9, respectively. DNA binding constant, K_b_*,* was determined by dividing the slope by the *Y* intercept. The K_b_ values were evaluated as 0.933 × 10^5^ M^−1^ and 3.08 × 10^5^ M^−1^ for the Schiff base ligand (L) and Cu–L bulk complex, respectively, indicating higher binding affinity for the copper complex. The intrinsic binding constant (K_b_) of the Cu(II) complex is comparable to those reported for classical DNA intercalators such as ethidium bromide (10^5^–10^6^ M^−1^)^89^. In contrast, the free Schiff base ligand exhibits a lower binding constant (2.3 × 10^4^ M^−1^), indicating a weaker interaction with DNA. The enhanced binding affinity upon complexation may be attributed to increased planarity and π–π stacking interactions facilitated by metal coordination. These results suggest a predominantly intercalative mode of binding for the Cu(II) complex^90^.

##### Ethidium bromide displacement studies

Fluorescence quenching measurement is a useful method for monitoring and comparing how effectively small molecules bind to DNA and for determining the nature of that binding^91^. The conjugate planar structure of the fluorophore EB (ethidium bromide) exhibits very weak fluorescence on its own. However, in the presence of DNA, it shows strong emission at around 600 nm due to significant intercalation between adjacent DNA base pairs. The addition of another molecule can reduce this fluorescence emission, and both the ligand and its copper complex quenched the fluorescence of the DNA-EB system. The strong emission can be quenched by various minor groove agents^92^. Hence, it is deduced that the considered ligand and its complex could bind to the minor groove of DNA. The observed fluorescence quenching behavior followed the typical Stern–Volmer relationship^93^, as shown in Figs. S4 and S5. The Stern–Volmer constant (K_sv_) indicates the efficiency of fluorescence quenching, which reflects the binding strength of the compound with DNA. K_sv_ values were 0.391 × 10^5^ M^−1^ and 5.02 × 10^5^ M^−1^ for the ligand (L) and the Cu–L complex, respectively. These values are significantly greater than those reported for related compounds. The higher K_sv_ value confirms strong interaction and possible competitive intercalation with EB. 

##### Viscosity measurements

Viscosity measurements were performed on CT-DNA to determine the binding mode of the ligand and its copper complex. The concentrations of the ligand and complex were varied during these experiments. A classical intercalator significantly raises the DNA solution viscosity because the molecule inserts between base pairs, forcing them apart and lengthening the overall DNA molecule^94^. In contrast, partial or non-traditional intercalation by complexes may instead cause the DNA helix to bend, leading to shorter effective length and an accompanying reduction in viscosity^95^. The variations in the relative specific viscosity of CT-DNA in the absence and presence of ligand (L) and Cu–L complex were plotted against [Schiff base ligand (L)] or [Cu–L Complex]/[CT-DNA]. As presented in Fig. S6, an increase in DNA viscosity was observed upon increasing concentrations of both the ligand and the complex. Intercalation of the investigated molecules into the double-stranded DNA or binding to the phosphate group of the DNA backbone generally increases DNA viscosity^96^.

### Cytotoxicity

Cancer is one of the diseases that poses a major threat to human life and health. Conventional cancer treatment, such as surgery, chemotherapy, and radiotherapy, is intimidating for patients due to its side effects. However, there are several metal-based anti-tumor medicines available, but their degrees of toxicity frequently restrict their clinical efficacy and therapeutic outcomes. The development of anti-tumor medications with increased efficacy and decreased toxicity is still the main goal of pharmaceutical chemistry^97^. Cu–L complexes have the potential to treat cancer because of their capability to produce reactive oxygen species (ROS), bind to DNA, and inhibit important enzymes that promote tumor progression^98^.

In this study, human breast adenocarcinoma cells known as MCF-7 cells were used to investigate the cytotoxicity of the ligand and its nanosized Cu complex. To evaluate cytotoxicity, we used the methylthiazol tetrazolium (MTT) assay. The cytotoxic effects of L and Cu–L complex were evaluated by determining IC_50_ values, which were compared to the reference drug Cisplatin (IC_50_ = 5.68 μM). The concentration needed to prevent 50% cell growth is represented by the IC_50_ value. The Cu–L complex demonstrated a high potency with IC_50_ = 30.30 μM, which is lower than Cisplatin but significantly higher than the free ligand (IC_50_ = 192.10 μM) against MCF-7, indicating enhancement upon coordination. The MTT assay plots for the ligand (L), Cu–L complex, and Cisplatin are shown in Fig. S7.

### Computational details

#### Molecular orbital studies

**Fig. 10:**
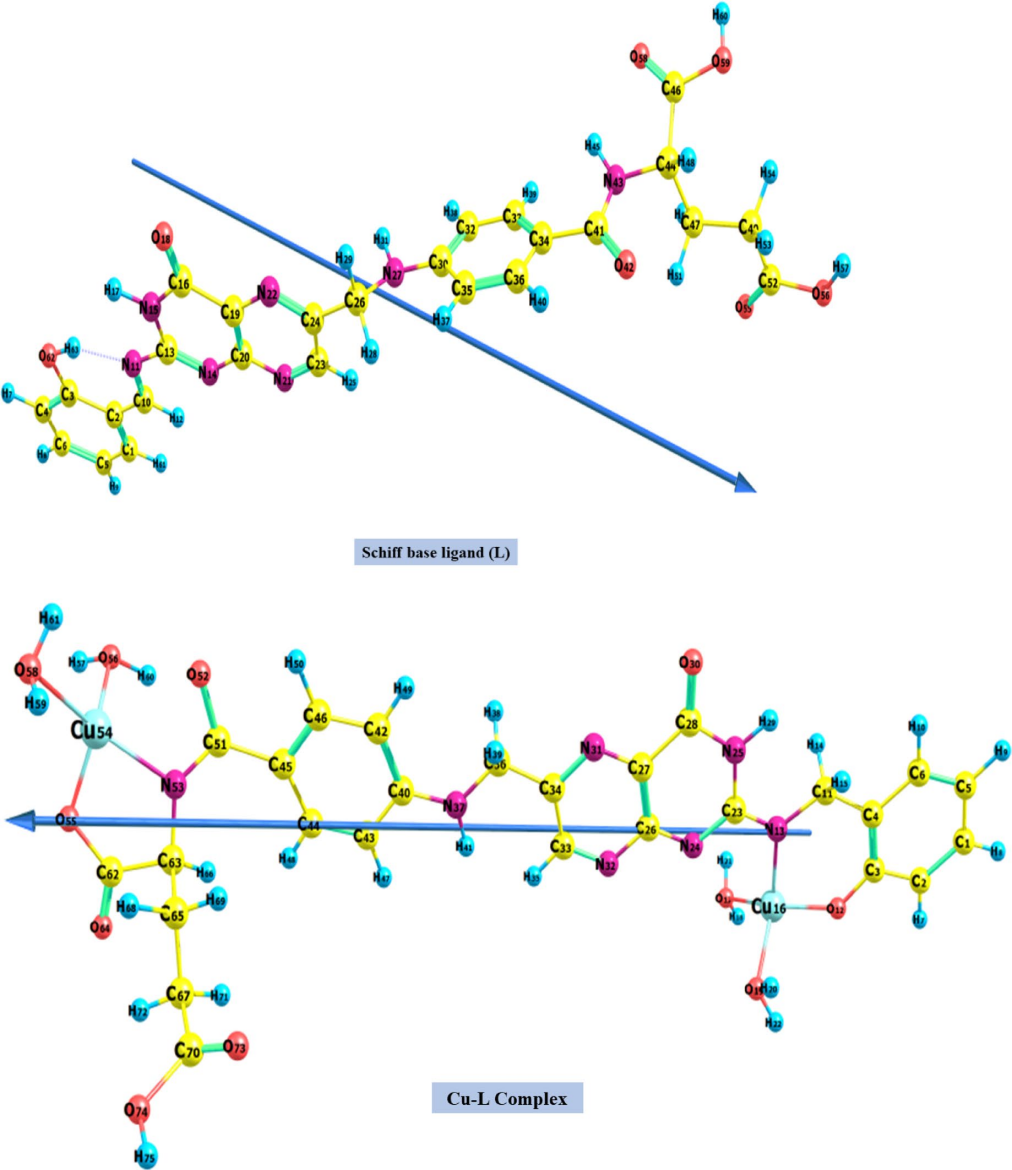
Optimized geometry, numbering system, and vector of dipole moment for the studied Schiff base ligand (L) and Cu–L complex using B3LYP/6-311G(d,p) and B3LYP/6-311G**-LANL2DZ level.

As seen in Fig. 10, the most stable structure of the Schiff base ligand (L) was identified through optimization using the B3LYP/6-311G(d,p) level of density functional theory. The dihedral angle values in Table S6, which vary from 0 to 180 degrees, show that the ligand (L) is not planar and emphasize its non-coplanar geometric structure. The estimated natural charges on the anticipated coordination sites listed in Table S7 can be used to determine the chelating centers of the ligand (L). Concerning the Schiff base ligand (L), the corresponding natural charges of the O62, O42, N11, and N43 atoms are -0.661, -0.649, -0.612, and -0.641, respectively. This highlights the potential of these atoms as chelation sites to bind to the Cu(II) cation.

#### Solid chelates geometry

The ground state geometry of the Cu–L complex solid chelate was optimized using the B3LYP/6-311G(d,p)-LANL2DZ mixed basis sets. The numbering scheme, optimized shape, and dipole moment vector of the Cu–L chelate are displayed in Fig. 10. Table S6 lists the values of bond lengths, bond angles, and dihedral angles of the complex surrounding the central metal ion. The C10-N11 bond in the Cu–L complex is observed to be longer than that in the Schiff base ligand (L), which can be attributed to the formation of coordinate bonds between the N11 atom and the metal center. Also, the bond lengths between C3 and O62 are shorter than in the ligand, indicating a center of chelation. The coordinate covalent bond in the metal-nitrogen complex is longer than normal M–N bond lengths, decreasing the chelate’s ionic character^99^. N11, N43, O42, and O62 of the ligand (L) moiety are the chelation sites in the Cu–L complex. According to the coordinates of N11, N43, O42, and O62, the bond angles indicate that the tetrahedral shape is the most stable for the Cu–L complex.

#### Global reactivity descriptors

Examining the spatial arrangement of molecular orbitals and energy levels is essential for designating electron density and charge movement in any chemical compound. The HOMO and LUMO mapping diagrams for the ligand (L) and its Cu–L complex are displayed in Fig. S8. HOMO and LUMO energies are necessary to determine the compounds’ redox potentials. Furthermore, the energy gap (E_gap_), defined as the energy difference between E_HOMO_ and E_LUMO_, is a crucial component in governing a molecule’s softness or hardness and evaluating its chemical reactivity. A smaller energy band gap indicates easier charge transfer and polarization, which are associated with higher global softness (S) and increased chemical reactivity, whereas larger E_gap_ values correspond to harder and less reactive molecules. Table 2 shows the computed and collected values for total energy, energy gap, electron affinity, ionization potential, electronegativity, chemical hardness, chemical potential, and global softness.

**Table 2:** Total energy, energy of HOMO and LUMO, energy gap, ionization energy (I), electron affinity (A), absolute electronegativities, ($\chi$), absolute hardness ($\eta$), global softness (S), chemical potential (V) of Schiff base ligand (L) and Cu–L complex using B3LYP/6-311G(d,p) and B3LYP/6-311G(d,p)-LANL2DZ level.

Parameter	Schiff base ligand (L)	Cu–L complex
E_T_, a.u	− 1915.02	− 2611.89
E_HOMO_, a.u	− 0.2182	− 0.1873
E_LUMO_, a.u	− 0.1107	− 0.1806
E_gap_, eV	2.9261	0.1820
I, eV	5.9384	5.0975
A, eV	3.0123	4.9155
χ, eV	4.4754	5.0065
η, eV	1.4630	0.0910
S, eV	0.3418	5.4931
V eV	− 4.4754	− 5.0065

The complex formation process destabilizes the HOMO of the Cu–L complex, as confirmed by the ionization potential (Ip) values. The E_HOMO_ is -0.1873 a.u. when compared to the Schiff base ligand (L) (-0.2182 a.u.). It was found, however, that the chelation process stabilizes the Cu–L complex’s LUMO. The HOMO is concentrated on some phenyl rings, oxygen, and nitrogen atoms. In the Schiff base ligand (L), the LUMO is distributed over some phenyl rings and the nitrogen atoms. The HOMO electron density is mainly located on the metal ion’s coordination sphere. In the Cu–L complex, the LUMO electron density is distributed across the copper cation moiety. Reactivity parameters listed in Table 2 highlight that the Cu–L complex has a lower E_gap_ value than the Schiff base ligand (L). This proposes that the Cu–L complex is highly reactive, with a higher softness (S) and a lower hardness (η); hence, polarization and charge transfer normally take place.

#### Molecular electrostatic potential (MESP)

Figure S9 presents the three-dimensional electrostatic potential (ESP) and molecular electrostatic potential (MESP) maps for the ligand (L) and its Cu–L complex. These maps were generated from optimized geometries using B3LYP/6-311G(d,p) and B3LYP/6-311G(d,p)-LANL2DZ for the ligand and its chelate, respectively. The LANL2DZ basis set was employed for the Cu(II) center in our DFT calculations because it is widely recognized for accurately treating transition metal complexes. It provides an effective core potential (ECP) that efficiently models both core and valence electrons while reducing computational cost. This basis set has been successfully applied in numerous studies on copper-containing Schiff base complexes, reproducing geometries, electronic structures, and spectroscopic properties in good agreement with experimental data. For instance, LANL2DZ has been used in the computational characterization of Cu(II) complexes for geometry optimization, electronic structure, and UV–Vis spectra predictions^49,50^. Thus, its selection is both computationally efficient and scientifically justified for the study of Cu(II) Schiff base complexes. Determining electrophilic or nucleophilic attack requires calculating the inherent charge on the active sites of every molecule, as well as charge transfer^100^. Red < orange < yellow < green < blue represents the color scheme of the potential^101,102^. The results demonstrate that the red (negative) regions are primarily localized over the nitrogen and oxygen atoms due to the presence of their lone electron pairs involved in coordination within the Cu–Schiff base complex. In contrast, blue (positive) potential sites are around some hydrogen and carbon atoms. The negative potentials of O42, O62, N11, and N43 in the ligand are confirmed by the yellow color at these positions. The chelate’s N and O atomic locations were observed to carry the maximum negative potential. Conversely, hydrogen atoms mostly represent positive areas. Atomic sites with negative electrostatic potential are typically susceptible to electrophilic attack. Consequently, greater negative potential increases the probability of electrophilic attack.

#### Natural charge and natural population

The electrical properties of any molecular system are mainly determined by analyzing the charge distribution over its atomic sites. The natural electronic configuration and atomic natural charges of the divalent Cu ion are listed in Table S7. Calculations revealed that the copper ion carries a charge lower than its formal (+ 2) charge because of the electron donation from the ligand’s O42, O62, N11, and N43 atoms. As shown in Table S7, the atoms O42, O62, N11, and N43 have the highest negative charge concentration, indicating that these atoms act as electron-donation sites of the ligand. However, the electropositive charge of the Cu 2:1 chelate is 0.729 and 0.811, which can be attributed to the Cu 3d^9.71^ and 3d^9.59^ orbitals. The metal ions in the Cu–L complex receive 1.271e (4d^9.71^) and 1.189e (3d^9.59^) from the ligand (L), as shown in Table S8.

#### Nonlinear optical properties (NLO)

The distribution of atomic charges is useful for determining the direction and magnitude of the dipole moment. Because of the widespread use of NLO compounds in optical information processing, data storage, and optical computing, they have attracted considerable attention^103,104^. Urea was chosen as a model NLO compound and reference, as the studied compounds lacked experimental NLO values. The results obtained are comparable to previous studies^105^. Table S9 displays the experimental value of urea^106^, along with the dipole moment, hyperpolarizabilities, and total static polarizabilities of the ligand and its complex, based on the similarity level of the applied theory. The polarizabilities and first-order hyperpolarizabilities were measured in atomic units (au). These values were multiplied by a conversion ratio of 8.6393 × 10^–33^ esu for (β) values and 0.1482 × 10^–24^ esu for (α) values. Table S9's data indicate that L and Cu–L compounds are polar, as indicated by their dipole moments (μ). Consequently, the dipole moments of the Cu–L complex are larger than those of urea, indicating higher polarity compared to the ligand (L). One of the most significant characteristics of the NLO system is the first-order hyperpolarizability factor. The β values show that the Schiff base ligand (L) is 19 times that of urea, while the Cu–L complex is 30 times that of urea.

### Molecular docking

**Fig. 11:**
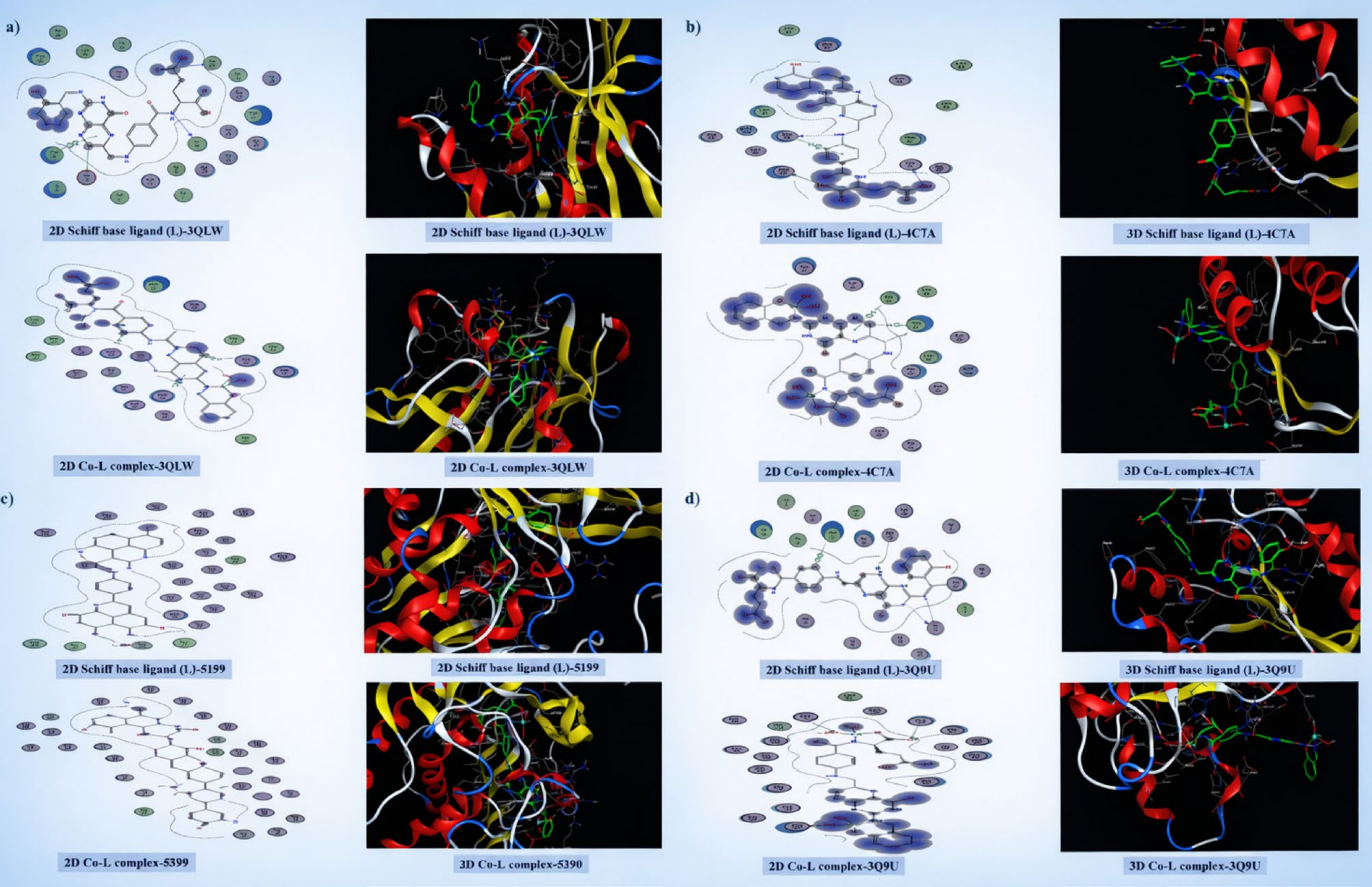
Molecular docking simulation studies of the interaction between the studied Schiff base ligand (L) and its Cu–L complex and the active site of the receptor of Aspergillus niger (**a**), Candida albicans (**b**), *Escherichia coli* (**c**), and *Staphylococcus aureus* (**d**).

Inhibiting cell wall, DNA, and protein synthesis, as well as metabolism, are mechanisms of action for antibacterial drugs^107^. Molecular docking studies validated the inhibitory potency of the tested compounds. Based on experimental bacterial activity results, bacterial binding pocket residues were selected from the PDB to analyse their binding profile with the target protein. *A. niger, C. albicans, E. coli,* and *S. aureus* were the four distinct bacteria that were docked with all synthesized compounds. They were examined for their interactions with the protein receptor and binding free energy. The docking data in Table 3 provide insight into the binding interactions (in kcal/mol) between the synthesized compounds and bacterial protein receptors. The two- and three-dimensional illustrations of docking interactions for all bacterial cells are shown in Fig. 11, where hydrogen bonds are represented as dashed lines. It was noted that the binding interaction surfaces of the synthesized compounds with bacterial protein receptors involved pi‑hydrogen, hydrogen‑donor, hydrogen‑pi, hydrogen‑acceptor, pi‑pi, and metal interactions. The negative binding affinities show the occurrence of these processes. A reduction in the binding energy of the examined materials toward the protein receptor reinforces their binding affinity. The data in Table 3 showed that the Cu–L complex has a more negative binding energy score with the 5I39 protein receptor. The binding affinity of Cu–L complex-5I39 > Cu–L complex-3QLW > Cu–L complex-3Q8U > Cu–L complex-4C7A chelates indicates the higher inhibition effect of Cu–L complex-5I39 against *Escherichia coli* (PDB ID: 5I39). According to the results obtained from the theoretical studies, we can conclude that the Cu complex under study has a small (η) value, which shows that it can transfer charge to the protein receptors. As a result, the 2:1 Cu complexes show an increasing sequence of charge transfer.

**Table 3:** The Docking interaction data calculations of the Schiff base ligand (L) and Cu–L complex with the active site of the receptor of *Aspergillus niger* (PDB ID: 4C7A), *Candida albicans* (PDB ID: 3QLW), *Escherichia coli* (PDB ID: 5I39), and *Staphylococcus aureus* (PDB ID: 3Q8U).

System	Binding score (kcal/mol)	Receptor	Interaction	Distance (Å)	E (kcal/mol)
*Aspergillus niger* (PDB ID: 4C7A)					
Schiff L-4C7A					
N 32	− 7.05	O THR 55	H-donor	2.86	− 1.2
O 55		OD1 ASN 54	H-donor	2.96	− 1.7
O 61		O LYS 52	H-donor	3.00	− 2.5
6-ring		CB THR 55	pi-H	3.60	− 0.6
Cu–L Complex-4C7A					
C 16	− 6.82	6-ring PHE 81	H-pi	4.19	− 0.8
C 20		6-ring PHE 81	H-pi	4.34	− 0.5
6-ring		CA ALA 85	pi-H	3.81	− 0.5
*Candida albicans* (PDB ID: 3QLW)					
Schiff L-3QLW					
C 26	− 9.39	OE1 GLU 32	H-donor	3.36	− 0.8
N 46		O ILE 19	H-donor	3.07	− 1.0
O 63		N ALA 115	H-acceptor	3.22	− 2.0
6-ring		6-ring PHE 36	pi-pi	3.84	− 0.0
Cu–L Complex-					
3QLW	− 8.75	N THR 58	H-acceptor	2.97	− 1.7
O 31		N GLY 114	H-acceptor	3.10	− 0.7
O 31		CB LYS 57	pi-H	3.92	− 0.8
6-ring		CD LYS 57	pi-H	4.07	− 1.0
6-ring		OG1 THR 58	pi-H	3.56	− 1.1
6-ring		N GLU 116	pi-H	4.30	− 2.2
*Escherichia coli* (PDB ID: 5I39)					
Schiff L-5I39					
O 23	− 10.78	SE MSE 440	H-donor	3.25	− 2.4
O 23		CA SER 93	H-acceptor	3.10	− 0.5
O 23		OG SER 93	H-acceptor	2.95	− 1.3
Cu–L Complex-5I39					
Cu 34	− 12.68	OE1 GLU 84	Metal	2.43	− 0.9
*Staphylococcus aureus* (PDB ID: 3Q8U)					
N 17	− 8.89	O GLY 116	H-donor	3.11	− 1.6
N 15		N GLY 116	H-acceptor	3.21	− 3.0
N 22		NH1 ARG 102	H-acceptor	3.20	− 1.7
N 22		NH2 ARG 102	H-acceptor	2.91	− 2.2
N 28		ND2 ASN 112	H-acceptor	3.54	− 1.3
6-ring		6-ring PHE 57	pi-pi	3.50	− 0.0
Cu–L Complex-3Q8U					
Cu 34	− 8.06	OE2 GLU 121	H-donor	2.65	− 0.5
O 68		ND1 HIS 115	H-donor	3.11	− 2.4
O 56		OG1 THR 91	H-acceptor	2.74	− 1.9
O 56		NH1 ARG 102	H-acceptor	2.80	− 1.8
O 64		NH1 ARG 85	H-acceptor	3.15	− 1.5
O 68		NZ LYS 9	H-acceptor	2.94	− 4.0
O 70		NZ LYS 9	H-acceptor	2.95	− 1.6

## Conclusions

In summary, the study describes the deliberate design and synthesis of a novel Schiff base ligand (L) from folic acid and salicylaldehyde, along with its Cu(II) complex (Cu–L) prepared in both bulk and nanoscale forms, employing a controlled 2:1 metal-to-ligand ratio. Comprehensive structural characterization, supported by DFT calculations, confirmed the successful formation and stability of the compounds. Biological evaluation revealed that Cu–L exhibits markedly enhanced activity compared to the free ligand, demonstrating potent antimicrobial effects against *Escherichia coli*, *Staphylococcus aureus*, and *Candida albicans*, high DNA-binding affinity (K_b_ = 3.08 × 10^5^ M^−1^), and significant cytotoxicity against the MCF-7 breast cancer cell line (IC₅₀ = 30.30 µg/mL). These findings underscore the critical role of Cu(II) coordination in augmenting the biological efficacy of the Schiff base ligand, positioning Cu–L as a promising multifunctional agent with potential applications in antimicrobial therapy and anticancer drug development.

## Supplementary Information

Below is the link to the electronic supplementary material.

Supplementary Material 1

## Data Availability

All data are included in this published article and its supplementary information file.
