# Supplementary material for: Synthesis, spectroscopic, characterization, antimicrobial, DNA interaction, DFT and molecular docking studies of a new Cu(II)-Schiff base complex
Source: Sci Rep. 2026 Apr 28;16:13636. doi: 10.1038/s41598-026-44842-5 (PMC13125568; doi:10.1038/s41598-026-44842-5)
Supplement: Supplementary file 1 — Supplementary Material 1 [file 41598_2026_44842_MOESM1_ESM.docx]

**Synthesis, spectroscopic, characterization, antimicrobial, DNA interaction, DFT and molecular docking studies of a new Cu(II)- Schiff base complex**

**Eman G. Mahmoud^a^, Eman H. Ismail^a^, Ayman A. Abdel Aziz^a^,** **Samir A. Abdel-Latif^b^, Sarah N. Mobarez^a,*^**

^a^Chemistry Department, Faculty of Science, Ain Shams University, Abbassia, Cairo 11566, Egypt

^b^Chemistry Department, Faculty of Science, Capital University (formerly Helwan University), Cairo 11795, Egypt

**Table of Contents**

| **Instrumentation** | **S-4** |
| --- | --- |
| **Biological efficiency** | **S-5** |
| **Antimicrobial activity** | **S-5** |
| **DNA binding experiments** | **S-5** |
| **Cytotoxicity studies** | **S-7** |
| **Table S1.** FT-IR spectral data of the starting material (folic acid and salicylaldehyde), the prepared Schiff base ligand (L), and its Cu–L complex in both bulk and nanosized forms. | **S-8** |
| **Fig. S1.** ESR spectrum of Cu–L bulk complex. | **S-9** |
| **Table S2.** Some fragmentations for Schiff base ligand (L) | **S-9** |
| **Table S3.** ^1^H –NMR data of Schiff base ligand (L) | **S-10** |
| **Table S4.** ^13^C NMR data of Schiff base ligand (L) | **S-11** |
| **Fig. S2.** Chromatogram of Cu–L bulk complex  **Table S5.** Thermogravimetric analysis (TGA) data obtained for Schiff base ligand (L) and its Cu–L complex in bulk and nanosized. | **S-12**  **S-12** |
| **Fig. S3.** TG-DTG curves of the Schiff base ligand (L), Cu–L bulk complex, and Cu–L nano complex | **S-14** |
| **Fig. S4.** Emission spectra of EB-CT-DNA (1 × 10^-4^ M, 1:1 molar ratio) in the absence (dashed line) and presence (solid lines) of Schiff ligand (L) (0–50 μM) at 25 °C and Plot of F_0_/F vs. [Schiff ligand (L)], where F_0_, F are the emission intensities of EB-DNA in the absence and presence of Schiff base ligand (L), respectively. | **S-15** |
| **Fig. S5.** Emission spectra of EB-CT-DNA (1 × 10^-4^ M, 1:1 molar ratio) in the absence (dashed line) and presence (solid lines) of Cu–L bulk complex (0–50 μM) at 25 °C and plot of F_0_/F vs. [Cu–L bulk complex], where F_0_, F are the emission intensities of EB-DNA in the absence and presence of Cu–L bulk complex, respectively. | **S-15** |
| **Fig. S6.** Effect of increasing Schiff base ligand (L) and Cu–L complex concentrations on the relative viscosity of CT-DNA at 25 ± 0.1 ^o^C. | **S-16** |
| **Fig. S7.** MTT assay for Schiff base ligand (L), Cu–L nanosized complex, and cisplatin. | **S-16** |
| **Table S6.** Selected geometric bond lengths, bond angles, and dihedral angles of the optimized Schiff base ligand (L) and Cu–L complex using B3LYP/6-311G(d,p) and B3LYP/6-311G(d,p)-LANL2DZ level | **S-17** |
| **Table S7.** NBO charges calculated for the Schiff base ligand (L) and Cu–L complex using B3LYP/6-311G(d,p) and B3LYP/6-311G(d,p) -LANL2DZ level  **Table S8.** Calculated natural population, natural charge, and natural electronic configuration of the metal in the studied Cu–L complex using B3LYP/6-311G**-LANL2DZ level.  **Fig. S8.** HOMO and LUMO maps for the Schiff base ligand (L) and Cu–L complex using B3LYP/6-311G(d,p) and B3LYP/6-311G(d,p)-LANL2DZ level.  **Fig. S9.** Molecular electrostatic potential (a) and contours of electrostatic potential surfaces (b) of the studied Schiff base ligand (L) and Cu–L complex operating B3LYP/6-311G(d,p) and B3LYP/6-311G(d,p)-LANL2DZ level. | **S-18**  **S-18**  **S-19**  **S-20** |
| **Table S9.** Calculated total static dipole moment (μ), the mean polarizability <α>, anisotropy of the polarizability Δα, and the first‐order hyperpolarizability <β > configuration for the Schiff base ligand (L) and Cu–L complex using B3LYP/6-311G** and B3LYP/6-311G**-LANL2DZ level. | **S-21** |
| **References** | **S-22** |

**Instrumentation**

Elemental analysis of carbon, hydrogen, and nitrogen was carried out using an automatic CHNS analyzer (Vario EL III, Elementar, Germany). FT-IR spectra were acquired using Thermo Scientific Nicolet iS10 Fourier Transform Infrared Spectrometer with KBr discs in the 4000–400 cm⁻¹ range. ^1^H NMR and ^13^C NMR spectra of the ligand were obtained using a Varian 400 MHz NMR spectrometer (USA), using DMSO-d₆ as a solvent and tetramethylsilane as an internal standard. UV-visible spectra were measured using a Shimadzu UV-Vis 1800 spectrophotometer and an Evolution 300 UV-visible spectrophotometer. Fluorescence measurements were conducted using a Jenway 6270 fluorimeter, with a pulsed xenon lamp as the excitation source. The metal contents were estimated by the atomic absorption spectroscopy (AAS) technique using a Savant AA Spectrophotometer according to the standard method APHA 3111B (2017) ^S1^. Electrospray ionization mass spectrometry (ESI-MS) analysis of Schiff base ligand (L) was carried out in positive ion mode on a XEVO TQD triple quadrupole instrument (Waters Corporation, Milford, MA, USA) using an ACQUITY UPLC BEH C18 column with a flow rate of 0.2 mL/min. Electron ionization mass spectrometry (EI-MS) analysis of the Cu(II) complex was carried out at 70 eV using an ISQ single quadrupole mass spectrometer (Thermo Scientific, USA/Italy, 2009). Thermal degradation of the compounds was studied using a Shimadzu TG-60H thermal analyzer under a nitrogen atmosphere using alumina as a reference. Magnetic moments were measured at room temperature (25 °C) using a Sherwood Scientific magnetic moment balance (model MK1), with Hg[Co(SCN)_₄_] as a calibrant. Diamagnetic corrections were determined using Pascal’s constants ^S2^. Electron spin resonance (ESR) measurements of the solid Cu(II) complexes were carried out at room temperature using a Bruker EMX EPR spectrometer at 9.5 GHz (X-band). The instrument operated with a microwave power of 1.0 mW and a modulation amplitude of 4.0 G. 2,2-Diphenylpyridylhydrazone (DPPH) was used as the standard (g = 2.0037). Transmission electron microscopy (TEM) analysis of the solid nanosized Cu(II) complex was performed using a JEOL JEM-100CX instrument. Gel permeation chromatography (GPC) analysis was done using a Shimadzu Nexera GPC system equipped with a highly sensitive refractive index detector. Dimethyl sulfoxide (DMSO) was used as a mobile phase, and the average molecular weight was calculated based on retention volume. The number average molecular weight (Mn), weight average molecular weight (Mw), and polydispersity index (PDI) were evaluated by instrument software.

**Biological efficiency**

**Antimicrobial activity**

The compounds were tested in vitro for their antibacterial activity against *Staphylococcus aureus* (Gram-positive bacteria) and *Escherichia coli* (Gram-negative bacteria) using nutrient agar medium. The antifungal activity was tested against *Candida albicans* and *Aspergillus niger* using Sabouraud dextrose agar medium. Ampicillin and Gentamicin were used as standard drugs for Gram-positive and Gram-negative bacteria, respectively. Nystatin was employed as a standard drug for fungal strains. DMSO was used as a solvent (negative) control. The concentration used to test the compounds against both bacterial and fungal strains was 15 mg/ml. The sterile media were added to the sterilized Petri dishes (20–25 mL per dish) and left to solidify at room temperature. The microbial suspension was prepared in sterile saline equivalent to McFarland 0.5 standard solution (1.5 x 10^5^ CFU mL^-1^), and its turbidity was set to an optical density (OD) of 0.13 using a spectrophotometer at 625 nm. Ideally, within 15 minutes of adjusting the turbidity of the inoculum suspension, a sterile cotton swab was dipped into the suspension and was used to flood the dried agar surface for 15 minutes while the lid was in place. Using a sterile borer, wells 6 mm in diameter were made in the solidified media. Using a micropipette, 100 μL of the tested compound's solution was added to each well, and the plates were kept at 37 °C for 24 hours in the event that antibacterial activity was detected. This experiment was performed in triplicate, and zones of inhibition were measured on a mm scale.

**DNA binding experiments**

In order to study the in vitro CT-DNA interaction with the Schiff base ligand (L) and its Cu–L complex, both were initially dissolved in DMSO (1 mM). Following that, these solutions were mixed with a 5 mM Tris-HCl-50 mM NaCl buffer solution (pH 7.4). The final concentration of DNA solutions used in the experiments never exceeded 1% DMSO (v/v). The solution of calf thymus DNA (CT-DNA) gave a ratio of UV absorbance at 260 and 280 nm, A_260_/A_280_, of 1.89, suggesting that the DNA was sufficiently free of protein. The concentration of CT-DNA was calculated from its absorption intensity at 260 nm with a molar extinction coefficient of 6600 M^−1^ cm^-1 S3^.

UV-Vis absorption titration experiments were carried out with the concentration of CT-DNA kept constant (10 µM), while the concentration of the Schiff base ligand (L) or its complex was varied from 0 to 90 mM. The mixtures were incubated at room temperature for approximately 10 min before the measurements. Control experiments with DMSO were conducted, and no influence on the spectra was noticed. From the absorption titration data, the binding *constant (K_b_)* was determined using the Wolfe-Shimer equation^S4^:

| $\frac{\mathbf{[ L or Cu-L]}}{\mathbf{(}\boldsymbol{\varepsilon}_{\mathbf{a}}\mathbf{-}\boldsymbol{\varepsilon}_{\mathbf{f}}\mathbf{)}}\mathbf{=}\frac{\mathbf{1}}{\mathbf{K}_{\mathbf{b}}\mathbf{(}\boldsymbol{\varepsilon}_{\mathbf{b}}\mathbf{-}\boldsymbol{\varepsilon}_{\mathbf{f}}\mathbf{)}}\mathbf{+}\frac{\mathbf{[L or Cu-L]}}{\mathbf{(}\boldsymbol{\varepsilon}_{\mathbf{b}}\mathbf{-}\boldsymbol{\varepsilon}_{\mathbf{f}}\mathbf{)}}$ | $\boldsymbol{(1)}$ |
| --- | --- |

where *ε_a_*, *ε_f_*, and *ε_b_* are the apparent, free, and bound complex extinction coefficients, respectively. *ε_f_* was evaluated using a calibration graph of the isolated metal complex in aqueous solution, following Beer's law. *ε_a_* was calculated as the ratio of the obtained absorbance to the compound concentration*,* A*_obs_ /* [L or Cu–L]. A plot of [L or Cu–L] / (*ε_a_*–*ε_f_*) versus [L or Cu–L] gave a line with a slope of 1 */ (*ε_b_*−*ε_f_*)* and a *Y* intercept equal to 1 */* K_b_*(*ε_b_*−*ε_f_*).* *K_b_* was determined as the ratio of the slope to the Y intercept.

The interaction of both L or Cu–L with CT-DNA has also been studied by tracking the changes noticed in the fluorescence intensity of the ethidium bromide DNA (EB-DNA) system. EB competitive experiments were done at room temperature by a stepwise addition of different concentrations of the Schiff base ligand (L) or its complex (0–50 µM) to the EB-bound CT-DNA solution (each of 1 × 10^-4^ M concentration, 1:1 molar ratio). The effect of the addition of each compound to the EB-DNA complex solution was studied by acquisition of the changes in the fluorescence emission spectra at an excitation wavelength of 520 nm, after incubation for ~ 10 min at room temperature. The measurements were repeated at least three times until there was no spectral change, showing that the binding or quenching had been achieved. The fluorescence spectra were investigated according to the classical Stern-Volmer equation ^S5^:

| $\frac{\mathbf{F}_{\mathbf{o}}}{\mathbf{F}}\mathbf{=1+}\mathbf{K}_{\mathbf{SV}}\left[ L or Cu-L \right]$ | $\boldsymbol{(2)}$ |
| --- | --- |

Further analysis for the type of interaction between the L or Cu–L and CT-DNA was conducted using a hydrodynamic method based on viscosity measurements. Viscosity was measured with an Ostwald viscometer immersed in a thermostatic water bath maintained at 25.0 ± 0.1 °C. The flow times (t) were noted for different concentrations of the ligand and its complex, while keeping the concentration of CT-DNA constant (50 µM). The buffer flow time in seconds was noted as *t_0_*. The viscosity coefficients for CT-DNA solutions in the absence (*η_0_*) and presence (*η*) of the ligand or its complex were calculated using the relation:

| $\boldsymbol{\eta=}\frac{\left( \mathbf{t-}\mathbf{t}_{\mathbf{0}} \right)}{\mathbf{t}_{\mathbf{0}}}$ | (3) |
| --- | --- |

The evaluation of the viscosity (η) of the samples was based on three replicate measurements. Data are presented as (η/η_0_) ^⅓^ versus the binding ratio of [Schiff base ligand (L) or Schiff complex] / [DNA[ ^S6^, where η and η_0_ are the viscosity coefficients of CT-DNA solutions in the presence and absence of the Schiff base ligand (L) or its complex, respectively.

**Cytotoxicity studies**

Tumor cell lines were grown in RPMI-1640 medium supplemented with 10% heat-inactivated fetal calf serum and 50 µg/mL gentamicin. The cells were kept at 37 °C in a humid atmosphere containing 5% CO₂ and sub-cultured two to three times weekly. For cytotoxicity assessment, cells were seeded at a density of 5 × 10⁴ cells per well in 96-well plates and incubated for 24 hours. Test compounds were added in twelve different concentrations, each in triplicate. Six wells with only media served as vehicle controls. After another 24-hour incubation, cell viability was evaluated using the MTT assay. The medium was replaced with 100 µL of phenol red-free RPMI-1640, and 10 µL of a 12 mM MTT stock solution (5 mg/mL in PBS) was added to each well, including controls. Plates were incubated for 4 hours at 37 °C and 5% CO₂. Subsequently, 85 µL of the medium was removed, and 50 µL of DMSO was added to solubilize the formazan crystals. After mixing and a 10-min incubation at 37 °C, absorbance was measured at 590 nm using a microplate reader. The percentage of cell viability was then calculated using the equation:

| **Cell viability % =** $\frac{\mathbf{OD}_{\mathbf{t}}}{\mathbf{OD}_{\mathbf{c}}}\boldsymbol{\times100}$ | **(4)** |
| --- | --- |

where ODₜ represents the average optical density of wells treated with the test compound, while OD_c_ corresponds to that of the untreated control cells. The relationship between cell viability and drug concentration was plotted to generate a survival curve for each tumor cell line following treatment. The IC₅₀ value, defined as the concentration of the compound that decreases cell viability by 50%, was obtained from the dose-response curves using GraphPad Prism software

(San Diego, CA, USA). This method is based on the colorimetric assay described previously, which enables rapid assessment of cell growth and cytotoxicity ^S7^.

**Table S1.** FT-IR spectral data of the starting material (folic acid and salicylaldehyde), the prepared Schiff base ligand (L), and its Cu–L complex in both bulk and nanosized forms.

| compound | ʋ(N-H)  primary | ʋ(N-H)  secondary | ʋ(O-H)  H_2_O | ʋ(O-H)  phenolic | ʋ(C=O) | ʋ(C=N)  imine | ʋ(C-N)aromatic  ʋ(C-N)aliphatic | ʋ(M-O) | ʋ(M-N) |
| --- | --- | --- | --- | --- | --- | --- | --- | --- | --- |
| Folic acid | 3322  3415 | 3240 | 3544 | - | 1693 | - | 1297.72  1160.55 | - | - |
| salicylaldehyde | - | - | - | 3178 | 1660  Aldehyde group | - | - | - | - |
| L | - | 3490 | - | 3257 | 1690 | 1537 | 1299.70  1239.08 | - | - |
| Cu–L (Bulk) | - | 3337 | 3224 | - | - | 1546 | 1337.66  1280.21 | 590  526 | 457  420 |
| Cu–L (Nano) | - | 3356 | 3268 | - | - | 1545 | 1338.63  1274.80 | 596  498 | 441  412 |


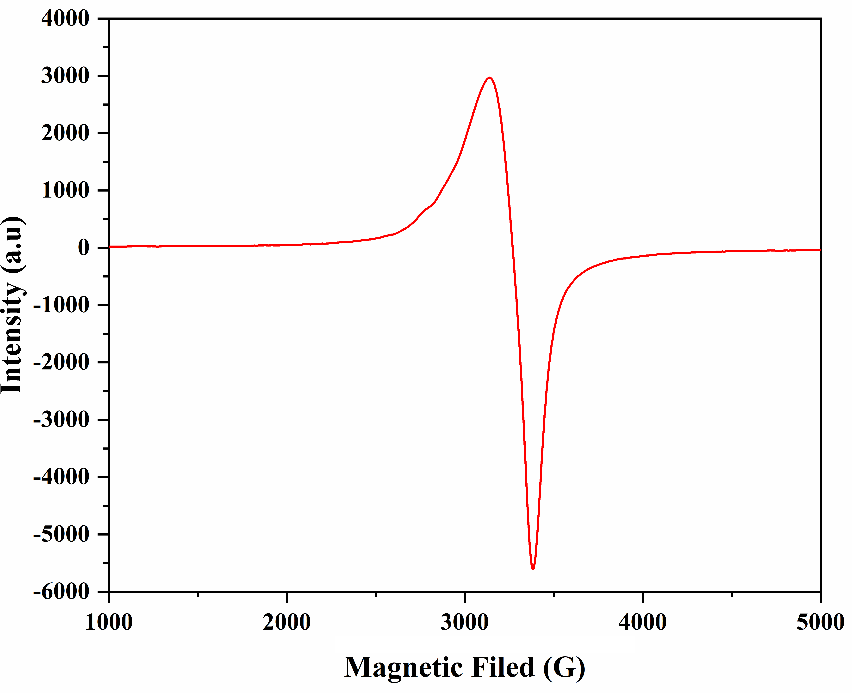


**Fig. S1.** ESR spectrum of Cu–L bulk complex.

**Table S2.** Some fragmentations for Schiff base ligand (L)

| fragmentations | Empirical formula | m/z |
| --- | --- | --- |
|  | [C_2_H_5_NO]^+^ | 59 |
|  + H^+^ | [C_3_H_5_O_2_ + H]^+^ | 74 |
|  - H^+^ | [C_7_H_5_N – H]^+^ | 102 |
|  | [C_7_H_7_O]^+^ | 107 |
|  + H_2_O | [C_7_H_6_N_2_O + H_2_O] ^+^ | 152 |
|  - 4H^+^ | [C_12_H_14_N_2_O_3_ – 4H]^+^ | 230 |
|  | [C_13_H_9_N_5_O_2_ + 7H]^+^ | 274 |
|  | [L + Hydroxyethylideneamine ion – 3H_2_]^+^ | 597 |
|  | [ (2 L + K + H_2_O]^+^ | 1149 |

**Table S3.** ^1^H –NMR data of Schiff base ligand (L)

| H No. | Type of H | δ (ppm) |
| --- | --- | --- |
| 1,2 | CH_2_ (q) | 1.89 |
| 3,4 | CH_2_ (t) | 2.28 |
| 5 | CH (t) | 4.28 |
| 6,7 | CH_2_ (s) | 4.41 |
| 8,9 | CH (d) | 6.54 |
| 10 | CH (d) | 6.63 |
| 11,12 | CH (t) | 6.9 |
| 13 | NH (s) | 7.08 |
| 14,15 | CH (d) | 7.58 |
| 16 | CH (d) | 7.62 |
| 17 | CH (s) | 8.62 |
| 18 | NH (s) | 8.67 |
| 19 | CH (s) | 9.03 |
| 20 | OH (s) | 10.84 |
| 21 | OH (s) | 12.7 |
| 22 | NH (s) | 12.7 |
| 23 | OH (s) | 12.7 |

**Table S4.** ^13^C NMR data of Schiff base ligand (L)

| C (No.) | δ (ppm) |
| --- | --- |
| 1 | 29.6 |
| 2 | 35.2 |
| 3 | 46.3 |
| 4 | 52.9 |
| 5,6 | 111.6 |
| 8 | 118.3 |
| 9 | 121 |
| 10 | 122 |
| 11 | 124 |
| 12,13 | 128.3 |
| 14,15 | 129.2 |
| 16,17,18 | 148.9 |
| 19 | 151.1 |
| 20 | 154.4 |
| 21 | 158 |
| 22 | 161.1 |
| 23 | 162.5 |
| 24 | 166.6 |
| 25 | 174.7 |
| 26 | 178 |


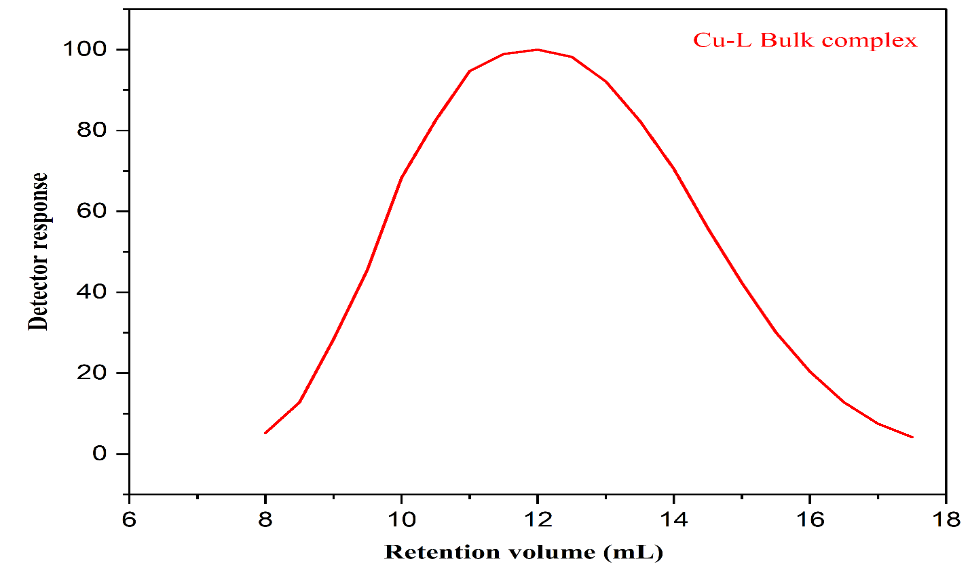


**Fig. S2.** Chromatogram of Cu–L bulk complex

**Table S5.** Thermogravimetric analysis (TGA) data obtained for Schiff base ligand (L) and its Cu–L complex in bulk and nanosized.

| Compound | Temp. range (°C) | Mass loss% | Assignments |
| --- | --- | --- | --- |
| Found Calc. | | | |
| Schiff base ligand (L) | 40-219  219-303  303-1000  1000 | 11.35 11.19  18.56 19.27  62.74 62.93  7.35 6.6 | CO_2_ + NH_3_  NH_3_ + CO_2_ + C_2_H_4_ +CH_4_  C_18_H_9_N_5_O_3_  Residue (3C) |
| Cu–L bulk | 48-154  154-208  208-357  357 – 887  Above 887 | 12.02 12.19  5.76 5.98  16.16 16.25  47.25 47.63  18.25 17.94 | 6H_2_O (hydrated)  2H_2_O(hydrated) + NH_3_  4H_2_O(coordinated) + CO_2_ + C_2_H_4_  C_23_H_14_N_6_O_3_  Residue (2CuO) |
| Cu–L nanosized | 38-149  149-268  268-519  Above519 | 10.2 10.16  15.5 16.14  50.76 50.34  23.46 23.36 | 5H_2_O(hydrated)  3H_2_O(hydrated)+ 4H_2_O(coordinated) + NH_3_  C_22_H_18_N_6_O_5_  Residue (2CuO + 4C) |


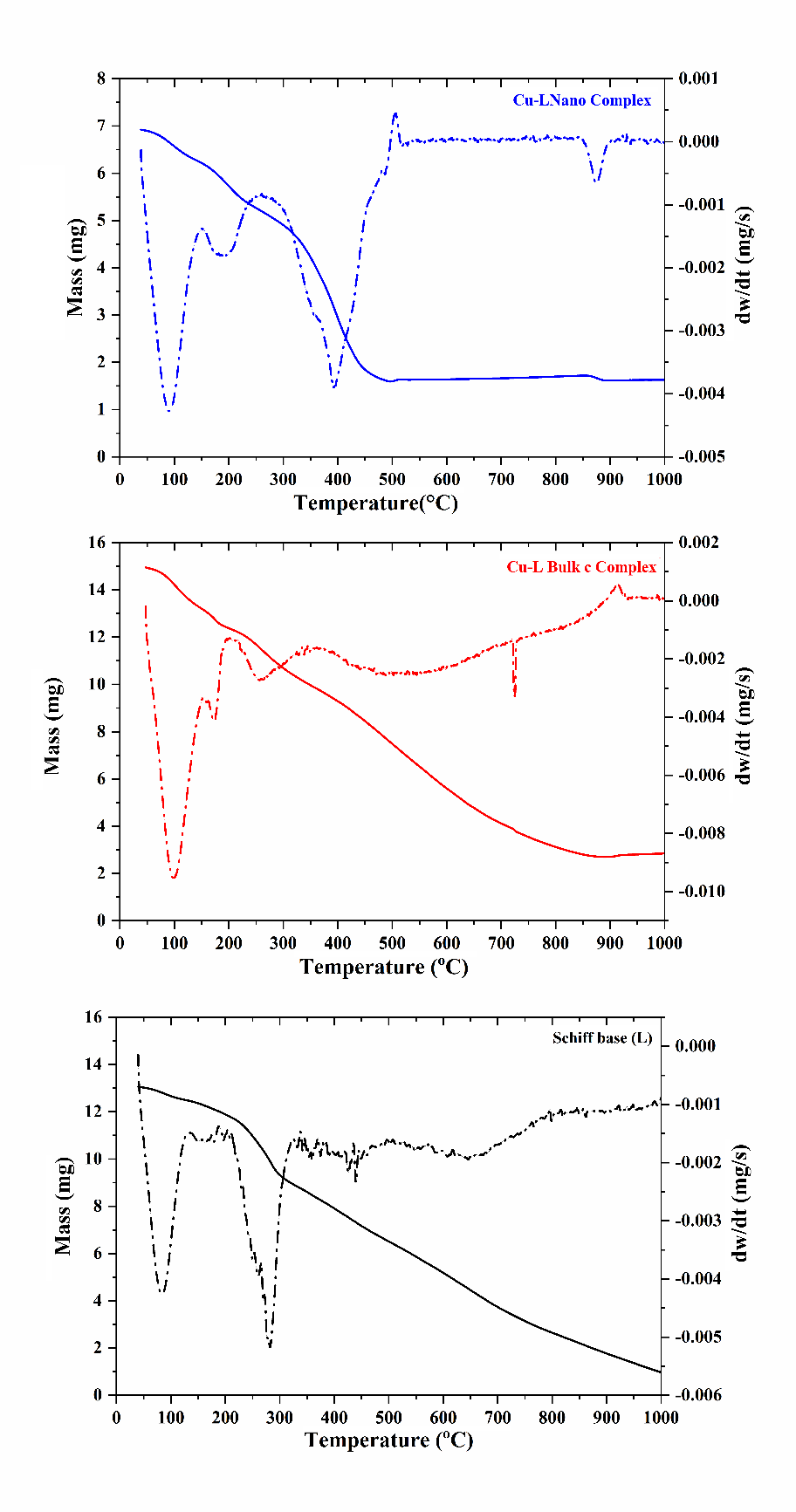


**Fig. S3.** TG-DTG curves of the Schiff base ligand (L), Cu–L bulk complex, and Cu–L nano complex.


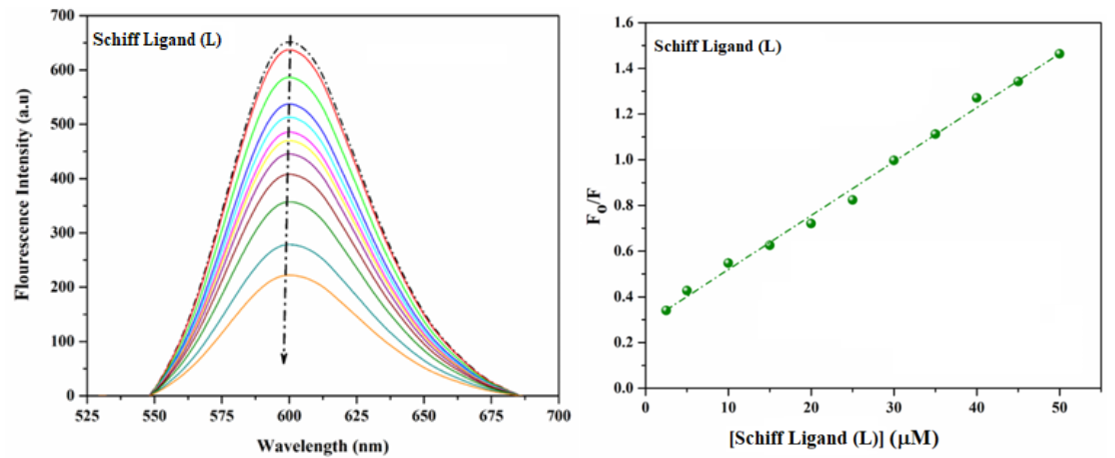


**Fig. S4**. Emission spectra of EB-CT-DNA (1 × 10^-4^ M, 1:1 molar ratio) in the absence (dashed line) and presence (solid lines) of Schiff ligand (L) (0–50 μM) at 25 °C and Plot of F_0_ / F vs. [Schiff ligand (L)], where F_0_, F are the emission intensities of EB-DNA in the absence and presence of Schiff base ligand (L), respectively.


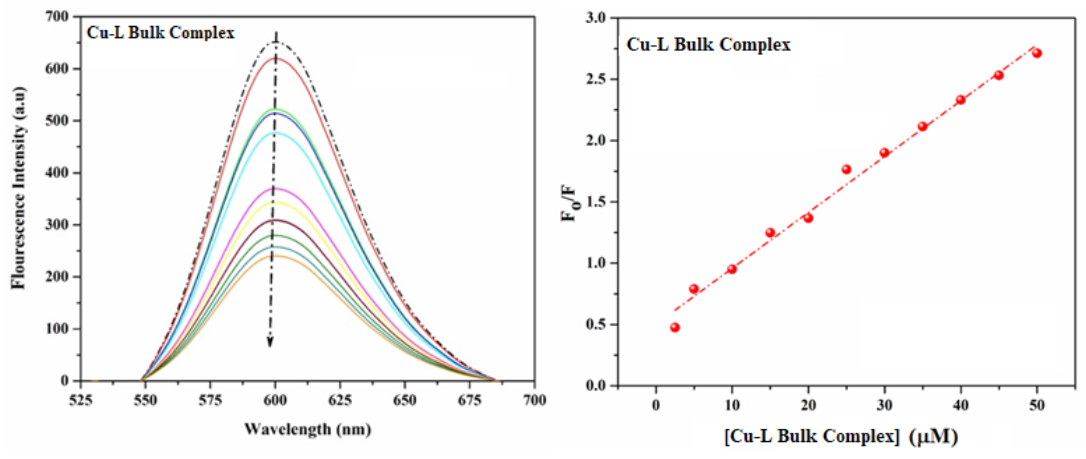


**Fig. S5.** Emission spectra of EB-CT-DNA (1 × 10^-4^ M, 1:1 molar ratio) in the absence (dashed line) and presence (solid lines) of Cu–L bulk complex (0–50 μM) at 25 °C and plot of F_0_ / F vs. [Cu–L bulk complex], where F_0_, F are the emission intensities of EB-DNA in the absence and presence of Cu–L bulk complex, respectively.


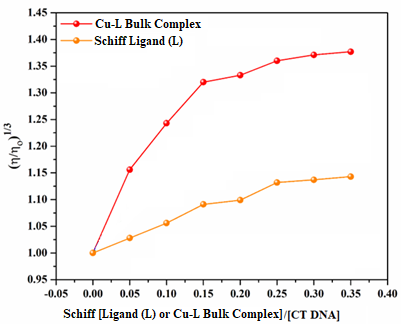


**Fig. S6.** Effect of increasing Schiff base ligand (L) and Cu–L Complex concentrations on the relative viscosity of CT-DNA at 25 ± 0.1 °C.


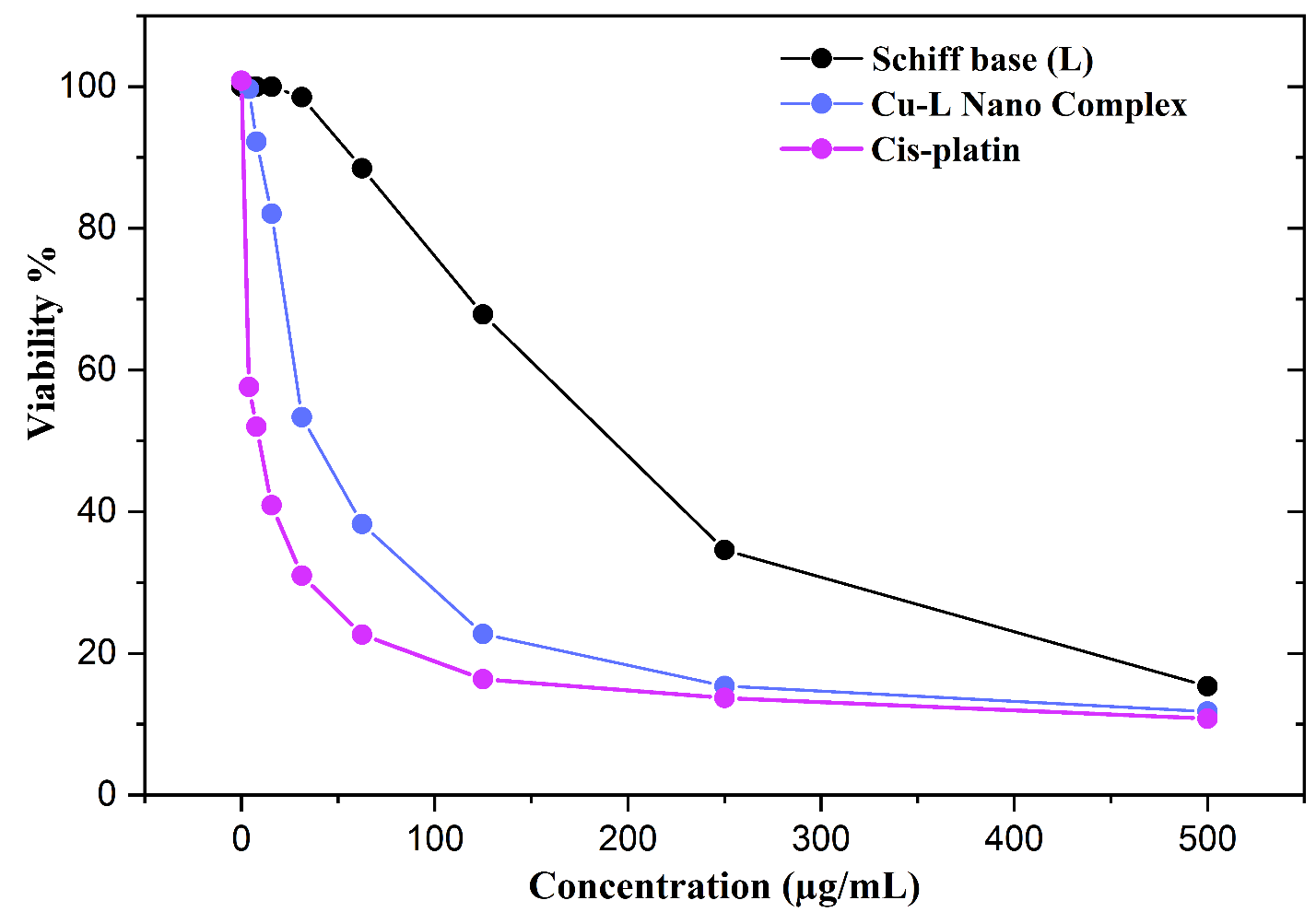


**Fig. S7.** MTT assay for Schiff base ligand (L), Cu–L nanosized Complex, and cisplatin.

**Table S6.** Selected geometric bond lengths, bond angles, and dihedral angles of the optimized Schiff base ligand (L) and Cu–L complex using B3LYP/6-311G(d,p) and B3LYP/6-311G(d,p)-LANL2DZ level.

| **Compound** | **Bond lengths (Å)** | | **Bond angles** | | **Dihedral angles** | |
| --- | --- | --- | --- | --- | --- | --- |
| Schiff base ligand (L) |  | | | | | |
|  | C6-C4 | 1.3846 | C6-C4-C3 | 120.136 | C6-C4-C3-O62 | -179.934 |
|  | C3-O62 | 1.3357 | O62-C3-C4 | 118.575 | O62-C3-C4-C6 | -179.934 |
|  | C1-C2 | 1.4129 | C1-C2-C10 | 119.246 | C1-C2-C10-N11 | -179.711 |
|  | C2-C10 | 1.4345 | C2-C10-N11 | 122.505 | C2-C10-N11-C13 | -179.968 |
|  | C10-N11 | 1.3010 | C10-N11-C13 | 118.004 | C10-N11-C13-N15 | -178.623 |
|  | C44-C46 | 1.5193 | C44-C46-O59 | 112.341 | O59-C46-C44-N43 | -8.159 |
|  | C46-C44 | 1.5193 | C46-C44-N43 | 35.298 | C46-C44-N43-C41 | 53.360 |
|  | C44-N43 | 1.4500 | C44-N43-C41 | 119.865 | C44-N43-C41-C34 | 176.065 |
|  | N43-C41 | 1.3719 | N43-C41-C34 | 116.651 | N43-C41-C34-C33 | 15.130 |
|  | C41-O42 | 1.2261 | O42-C41-C34 | 122.338 | O42-C41-C34-C33 | -164.519 |
| **Cu–L complex (2:1)** |  | | | | | |
|  | C2-C3 | 1.4233 | C2-C3-O12 | 116.642 | C2-C3-O12-Cu16 | 178.112 |
|  | C3-O12 | 1.3084 | C3-O12-Cu16 | 125.249 | C3-O12-Cu16-N13 | 0.619 |
|  | O12-Cu16 | 1.9221 | O12-Cu16-N13 | 99.538 | O12-Cu16-N13-C23 | -178.309 |
|  | N25-C23 | 1.3948 | N25-C23-N13 | 120.694 | N25-C23-N13-Cu16 | 177.566 |
|  | C23-N13 | 1.3340 | C23-N13-Cu16 | 118.299 | C23-N13-Cu16-O12 | -178.309 |
|  | C51-N53 | 1.3404 | C51-N53-Cu54 | 111.247 | C51-N53-Cu54-O55 | 175.894 |
|  | N53-Cu54 | 1.9348 | N53-Cu54-O55 | 86.695 | N53-Cu54-O55-C62 | 0.115 |
|  | C63-C62 | 1.5590 | C63-C62-O55 | 117.604 | C63-C62-O55-Cu54 | 3.425 |
|  | C62-O55 | 1.3096 | C62-O55-Cu54 | 114.003 | C62-O55-Cu54-N53 | 0.115 |

**Table S7.**  NBO charges calculated for the Schiff base ligand (L) and Cu–L complex using B3LYP/6-311G(d,p) and B3LYP/6-311G(d,p) -LANL2DZ level.

|  | Schiff Base Ligand (L) | Cu–L Complex |
| --- | --- | --- |
|  |  |  |
| O62  O42 | -0.661  -0.649 | -0.698  -0.699 |
| N11  N43 | -0.612  -0.641 | -0.653  -0.658 |
| Cu16  Cu54 | - | 0.729  0.811 |

**Table S8.** Calculated natural population, natural charge, and natural electronic configuration of the metal in the studied Cu–L complex using B3LYP/6-311G**-LANL2DZ level.

| Cu- chelate | Natural charge |  | Core | Natural population | | | Natural electronic configuration |
| --- | --- | --- | --- | --- | --- | --- | --- |
|  |  |  |  | **Valence** | **Rydberg** | **Total** |  |
| Cu16 | 0.729 |  | 17.9968 | 10.2624 | 0.0113 | 28.2705 | [core]4s^0.23^3d^9.71^4p^0.33)^5p^0.01^ |
| Cu45 | 0.811 |  | 17.9960 | 10.1816 | 0.0111 | 28.1887 | [core]4s^0.27^3d^9.59^4p^0.33^5p^0.01^ |

**
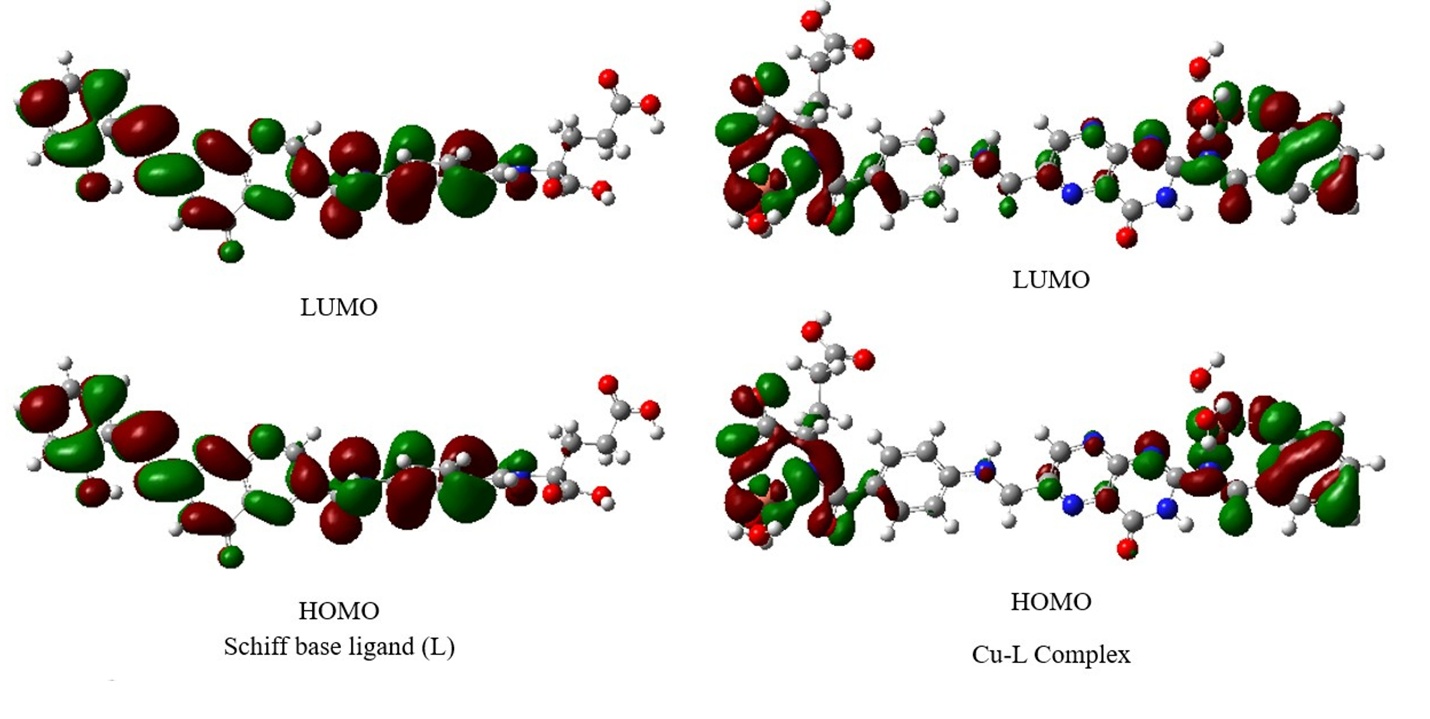
**

**Fig. S8.** HOMO and LUMO maps for the Schiff base ligand (L) and Cu–L complex using B3LYP/6-311G(d,p) and B3LYP/6-311G(d,p)-LANL2DZ level.


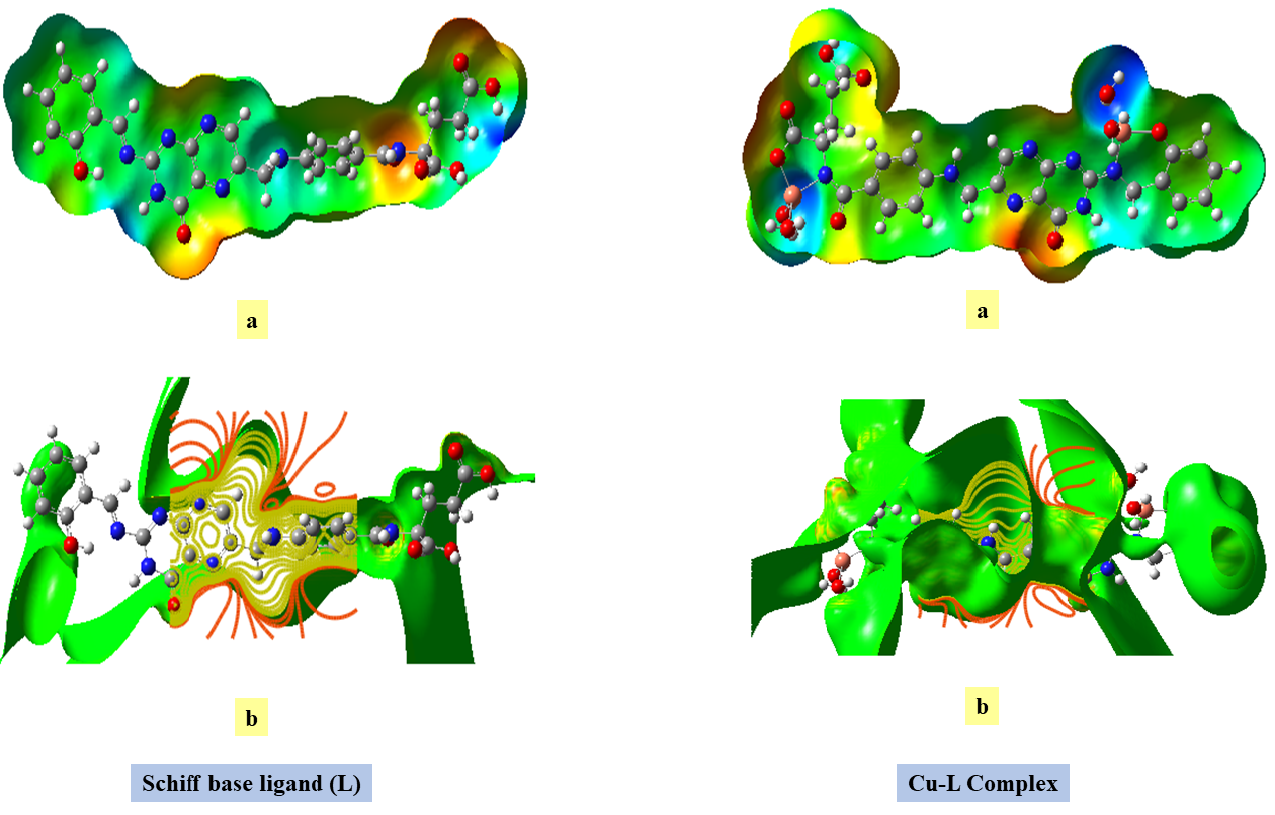


**Fig. S9.** Molecular electrostatic potential (a) and contours of electrostatic potential surfaces (b) of the studied Schiff base ligand (L) and Cu–L complex operating B3LYP/6-311G(d,p) and B3LYP/6-311G(d,p)-LANL2DZ level.

**Table S9.** Calculated total static dipole moment (μ), the mean polarizability <α>, anisotropy of the polarizability Δα, and the first‐order hyperpolarizability <β > configuration for the Schiff base ligand (L) and Cu–L complex using B3LYP/6-311G** and B3LYP/6-311G**-LANL2DZ level.

| Property | Urea | Schiff base ligand (L) | Cu–L complex |
| --- | --- | --- | --- |
| µ, D | 1.3197 | 4.83 | 7.47 |
| XX, a.u. | - | -157.4934 | -247.798 |
| YY | - | -256.5216 | -272.8765 |
| ZZ | - | -227.2859 | -250.6695 |
| XY | - | -71.5904 | 66.3508 |
| XZ | - | 11.7671 | 12.6741 |
| YZ | - | 1.8031 | -12.8717 |
| <α> esu | - | -3.1680x10^-23^ | -3.8104x10^-23^ |
| Δα, esu | - | 13.0602x10^-24^ | 3.5232x10^-24^ |
| XXX | - | -178.2146 | 710.3338 |
| XXY | - | -272.0253 | -438.9974 |
| XYY | - | -156.2082 | 9.8034 |
| YYY | - | 13.9787 | 169.2197 |
| XXZ | - | 198.4027 | 39.2321 |
| XYZ | - | 2.0648 | 100.8203 |
| YYZ | - | 27.3752 | -9.6072 |
| XZZ | - | 88.9087 | -102.6079 |
| YZZ | - | -13.2987 | 22.0974 |
| ZZZ | - | 0.4717 | 97.0984 |
| <β>, esu | 0.1947x10^-30^ | 3.7169x10^-30^ | 5.8514x10^-30^ |

**References**

S1. Rice, E. W., Baird, Rodger. & Eaton, A. D. Metals by flame atomic absorption spectrometry (3111B). In *standard methods for the examination of water and wastewater* (eds. Baird, Rodger., Eaton, A. D. & Rice, E. W.) 316–324 (American Public Health Association, American Water Works Association, Water Environment Federation, Washington, DC, 2017).

S2. Earnshaw, A. *Introduction to magnetochemistry*. (Academic Press, London, 1968).

S3. Alvarado, Y. J. *et al.* Thermodynamics of solution, interaction with calf thymus DNA and anticancer activity of phenylhydrazone derivatives. *J Solution Chem* **40**, 26–39 (2011).

S4. Jayasri, B., Rajeshwari, K., Vasantha, P. & Anantha Lakshmi, P. V. Ternary cobalt (II)-metformin-glycine/histidine/proline complexes: Multispectroscopic DNA, HSA, and BSA interaction and cytotoxicity studies. *Biol Trace Elem Res* **201**, 5481–5499 (2023).

S5. Hammam, M. M., Ramadan, R. M., Aziz, A. A. A., Sadek, M. A. & Salem, A. N. M. Novel heteroleptic ruthenium complexes incorporating 6,7-dichloro-2,3-di(pyridine-2-yl)quinoxaline as polypyridyl bridging ligand: Synthesis, characterization, photophysical, electrochemistry, in vitro biological activity and molecular docking studies. *J Mol Struct* **1323**, 140748 (2025).

S6. Elsheemy, W. M., Abdel Aziz, A. A., Ramadan, R. M., Kozakiewicz‐Piekarz, A. & Sayed, M. A. Crystal structure, Hirshfeld surface analysis, molecular modeling, electrochemical properties, and potential medicinal activity of a novel binuclear Co(II) complex. *Appl Organomet Chem* **38**, e7641 (2024).

S7. Mosmann, T. Rapid colorimetric assay for cellular growth and survival: Application to proliferation and cytotoxicity assays. *J Immunol Methods* **65**, 55–63 (1983).
